# A Formal C Memory Model for Separation Logic

**DOI:** 10.1007/s10817-016-9369-1

**Published:** 2016-05-10

**Authors:** Robbert Krebbers

**Affiliations:** 1 ICIS, Radboud University Nijmegen, Nijmegen, The Netherlands; 2 Aarhus University, Aarhus, Denmark

**Keywords:** ISO C11 standard, C verification, Memory models, Separation logic, Interactive theorem proving, Coq

## Abstract

The core of a formal semantics of an imperative programming language is a memory model that describes the behavior of operations on the memory. Defining a memory model that matches the description of C in the C11 standard is challenging because C allows both *high-level* (by means of typed expressions) and *low-level* (by means of bit manipulation) memory accesses. The C11 standard has restricted the interaction between these two levels to make more effective compiler optimizations possible, at the expense of making the memory model complicated. We describe a formal memory model of the (non-concurrent part of the) C11 standard that incorporates these restrictions, and at the same time describes low-level memory operations. This formal memory model includes a rich permission model to make it usable in separation logic and supports reasoning about program transformations. The memory model and essential properties of it have been fully formalized using the Coq proof assistant.

## Introduction

A memory model is the core of a semantics of an imperative programming language. It models the memory states and describes the behavior of memory operations. The main operations described by a C memory model are:

- Reading a value at a given address.
- Storing a value at a given address.
- Allocating a new object to hold a local variable or storage obtained via malloc.
- Deallocating a previously allocated object.

Formalizing the C11 memory model in a faithful way is challenging because C features both *low-level* and *high-level* data access. Low-level data access involves unstructured and untyped byte representations whereas high-level data access involves typed abstract values such as arrays, structs and unions.

This duality makes the memory model of C more complicated than the memory model of nearly any other programming language. For example, more mathematically oriented languages such as Java and ML feature only high-level data access, in which case the memory can be modeled in a relatively simple and structured way, whereas assembly languages feature only low-level data access, in which case the memory can be modeled as an array of bits.

The situation becomes more complicated as the C11 standard allows compilers to perform optimizations based on a high-level view of data access that are inconsistent with the traditional low-level view of data access. This complication has lead to numerous ambiguities in the standard text related to aliasing, uninitialized memory, end-of-array pointers and type-punning that cause problems for C code when compiled with widely used compilers. See for example the message [42] on the standard committee’s mailing list, Defect Reports #236, #260, and #451 [26], and the various examples in this paper.

*Contribution* This paper describes the $\mathrm{CH}_2\mathrm{O}$ memory model, which is part of the the $\mathrm{CH}_2\mathrm{O}$ project [30–37]. $\mathrm{CH}_2\mathrm{O}$ provides an operational, executable and axiomatic semantics in Coq for a large part of the non-concurrent fragment of C, based on the official description of C as given by the C11 standard [27].

The key features of the $\mathrm{CH}_2\mathrm{O}$ memory model are as follows:

- *Close to C11* $\mathrm{CH}_2\mathrm{O}$ is faithful to the C11 standard in order to be compiler independent. When one proves something about a given program with respect to $\mathrm{CH}_2\mathrm{O}$, it should behave that way with *any* C11 compliant compiler (possibly restricted to certain implementation-defined choices).
- *Static type system* Given that C is a statically typed language, $\mathrm{CH}_2\mathrm{O}$ does not only capture the dynamic semantics of C11 but also its type system. We have established properties such as type preservation of the memory operations.
- *Proof infrastructure* All parts of the $\mathrm{CH}_2\mathrm{O}$ memory model and semantics have been formalized in Coq (without axioms). This is essential for its application to program verification in proof assistants. Also, considering the significant size of $\mathrm{CH}_2\mathrm{O}$ and its memory model, proving metatheoretical properties of the language would have been intractable without the support of a proof assistant.
  Despite our choice to use Coq, we believe that nearly all parts of $\mathrm{CH}_2\mathrm{O}$ could be formalized in any proof assistant based on higher-order logic.
- *Executable* To obtain more confidence in the accuracy of $\mathrm{CH}_2\mathrm{O}$ with respect to C11, the $\mathrm{CH}_2\mathrm{O}$ memory model is executable. An executable memory model allows us to test the $\mathrm{CH}_2\mathrm{O}$ semantics on example programs and to compare the behavior with that of widely used compilers [33, 37].
- *Separation logic* In order to reason about concrete C programs, one needs a program logic. To that end, the $\mathrm{CH}_2\mathrm{O}$ memory model incorporates a complex permission model suitable for separation logic. This permission system, as well as the memory model itself, forms a separation algebra.
- *Memory refinements* $\mathrm{CH}_2\mathrm{O}$ has an expressive notion of memory refinements that relates memory states. All memory operations are proven invariant under this notion. Memory refinements form a general way to validate many common-sense properties of the memory model in a formal way. They also open the door to reasoning about program transformations, which is useful if one were to use the memory model as part of a verified compiler front-end.

This paper is an extended version of previously published conference papers at CPP [30] and VSTTE [32]. In the time following these two publications, the memory model has been extended significantly and been integrated into an operational, executable and axiomatic semantics [33, 37]. The memory model now supports more features, various improvements to the definitions have been carried out, and more properties have been formally proven as part of the Coq development.

Parts of this paper also appear in the author’s PhD thesis [33], which describes the entire $\mathrm{CH}_2\mathrm{O}$ project including its operational, executable and axiomatic semantics, and metatheoretical results about these.

*Problem* The C11 standard gives compilers a lot of freedom in what behaviors a program may have [27, 3.4]. It uses the following notions of under-specification:

- *Unspecified behavior*: two or more behaviors are allowed. For example: the execution order of expressions. The choice may vary for each use of the construct.
- *Implementation-defined behavior*: like unspecified behavior, but the compiler has to document its choice. For example: size and endianness of integers.
- *Undefined behavior:* the standard imposes no requirements at all, the program is even allowed to crash. For example: dereferencing a NULL pointer, or signed integer overflow.

Under-specification is used extensively to make C portable, and to allow compilers to generate fast code. For example, when dereferencing a pointer, no code has to be generated to check whether the pointer is valid or not. If the pointer is invalid (NULL or a dangling pointer), the compiled program may do something arbitrary instead of having to exit with a NullPointerException as in Java. Since the $\mathrm{CH}_2\mathrm{O}$ semantics intends to be a formal version of the C11 standard, it has to capture the behavior of *any* C compiler, and thus has to take *all* under-specification seriously (even if that makes the semantics complex).

Modeling under-specification in a formal semantics is folklore: unspecified behavior corresponds to non-determinism, implementation-defined behavior corresponds to parameterization, and undefined behavior corresponds to a program having no semantics. However, the extensive amount of underspecification in the C11 standard [27, Annex J], and especially that with respect to the memory model, makes the situation challenging. We will give a motivating example of subtle underspecification in the introduction of this paper. Section 3 provides a more extensive overview.

*Motivating example* A drawback for efficient compilation of programming languages with pointers is *aliasing*. Aliasing describes a situation in which multiple pointers refer to the same object. In the following example the pointers p and q are said to be *aliased*. 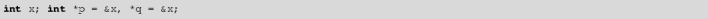


The problem of aliased pointers is that writes through one pointer may effect the result of reading through the other pointer. The presence of aliased pointers therefore often disallows one to change the order of instructions. For example, consider: 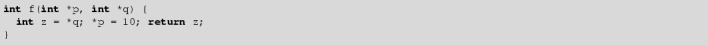


When f is called with pointers p and q that are aliased, the assignment to *p also affects *q. As a result, one cannot transform the function body of f into the shorter *p = 10; return (*q);. The shorter function will return 10 in case p and q are aliased, whereas the original f will always return the original value of *q.

Unlike this example, there are many situations in which pointers can be assumed *not* to alias. It is essential for an optimizing compiler to determine where aliasing cannot occur, and use this information to generate faster code. The technique of determining whether pointers can alias or not is called *alias analysis*.

In *type-based alias analysis*, type information is used to determine whether pointers can alias or not. Consider the following example: 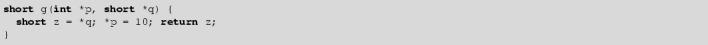


Here, a compiler is allowed to assume that p and q are not aliased because they point to objects of different types. The compiler is therefore allowed to transform the function body of g into the shorter *p = 10; return (*q);.

The peculiar thing is that the C type system does not statically enforce the property that pointers to objects of different types are not aliased. A union type can be used to create aliased pointers to different types: 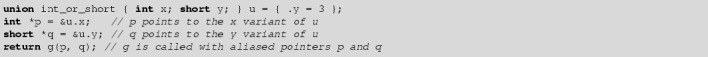


The above program is valid according to the rules of the C11 type system, but has undefined behavior during execution of g. This is caused by the standard’s notion of *effective types* [27, 6.5p6-7] (also called *strict-aliasing restrictions*) that assigns undefined behavior to incorrect usage of aliased pointers to different types.

We will inline part of the function body of g to indicate the incorrect usage of aliased pointers during the execution of the example.
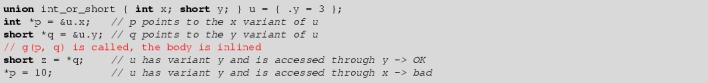


The assignment *p = 10 violates the rules for effective types. The memory area where p points to contains a union whose variant is y of type short, but is accessed through a pointer to variant x of type int. This causes undefined behavior.

Effective types form a clear tension between the low-level and high-level way of data access in C. The low-level representation of the memory is inherently untyped and unstructured and therefore does not contain any information about variants of unions. However, the standard treats the memory as if it were typed.

*Approach* Most existing C formalizations (most notably Norrish [45], Leroy et al. [39, 40] and Ellison and Roşu [19]) use an unstructured untyped memory model where each object in the formal memory model consists of an array of bytes. These formalizations therefore cannot assign undefined behavior to violations of the rules for effective types, among other things.

In order to formalize the interaction between low-level and high-level data access, and in particular effective types, we represent the formal memory state as a forest of well-typed trees whose structure corresponds to the structure of data types in C. The leaves of these trees consist of bits to capture low-level aspects of the language.

The key concepts of our memory model are as follows:

- *Memory trees* (Sect. 6.3) are used to represent each object in memory. They are abstract trees whose structure corresponds to the shape of C data types. The memory tree of struct S { short x, *r; } s = {33,&s.x } might be (the precise shape and the bit representations are implementation defined): 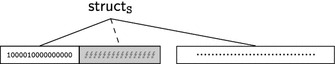 The leaves of memory trees contain permission annotated bits (Sect. 6.2). Bits are represented symbolically: the integer value 33 is represented as its binary representation 1000010000000000, the padding bytes as symbolic *indeterminate* bits  (whose actual value should not be used), and the pointer &s.x as a sequence of symbolic *pointer bits*.
  The *memory* itself is a forest of memory trees. Memory trees are explicit about type information (in particular the variants of unions) and thus give rise to a natural formalization of effective types.
- *Pointers* (Sect. 6.1) are formalized using paths through memory trees. Since we represent pointers as paths, the formal representation contains detailed information about how each pointer has been obtained (in particular which variants of unions were used). A detailed formal representation of pointers is essential to describe effective types.
- *Abstract values* (Definition 6.4) are trees whose structure is similar to memory trees, but have base values (mathematical integers and pointers) on their leaves. The abstract value of struct S { short x, *r; } s = { 33,&s.x } is: 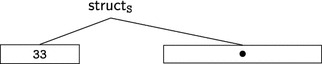 Abstract values hide internal details of the memory such as permissions, padding and object representations. They are therefore used in the external interface of the memory model and throughout the operational semantics.

Memory trees, abstract values and bits with permissions can be converted into each other. These conversions are used to define operations internal to the memory model. However, none of these conversions are bijective because different information is materialized in these three data types:

**Table:** 

	Abstract values	Memory trees	Bits with permissions
Permissions			
Padding		Always	
Variants of union			
Mathematical values			

This table indicates that abstract values and sequences of bits are complementary. Memory trees are a middle ground, and therefore suitable to describe both the low-level and high-level aspects of the C memory.

*Outline* This work presents an executable mathematically precise version of a large part of the (non-concurrent) C memory model.

- Section 3 describes some challenges that a C11 memory model should address; these include end-of-array pointers, byte-level operations, indeterminate memory, and type-punning.
- Section 4 describes the types of C. Our formal development is parameterized by an abstract interface to characterize implementation-defined behavior.
- Section 5 describes the permission model using a variant of separation algebras that is suitable for formalization in Coq. The permission model is built compositionally from simple separation algebras.
- Section 6 contains the main part of this paper, it describes a memory model that can accurately deal with the challenges posed in Sect. 3.
- Section 7 demonstrates that our memory model is suitable for formal proofs. We prove that the standard’s notion of effective types has the desired effect of allowing type-based alias analysis (Sect. 7.1), we present a method to reason compositionally about memory transformations (Sect. 7.2), and prove that the memory model has a separation algebra structure (Sect. 7.4).
- Section 8 describes the Coq formalization: all proofs about our memory model have been fully formalized using Coq.

As this paper describes a large formalization effort, we often just give representative parts of definitions. The interested reader can find all details online as part of our Coq formalization at: http://robbertkrebbers.nl/research/ch2o/.

## Notations

This section introduces some common mathematical notions and notations that will be used throughout this paper.

### **Definition 2.1**

We let $\mathbb {N}$ denote the type of *natural numbers* (including 0), let $\mathbb {Z}$ denote the type of *integers*, and let $\mathbb {Q}$ denote the type of *rational numbers*. We let  denote that $i \in \mathbb {N}$ is a *divisor* of $j \in \mathbb {N}$.

### **Definition 2.2**

We let $\mathsf{Prop}$ denote the type of *propositions*, and let $\mathsf{bool}$ denote the type of *Booleans* whose elements are $\mathsf{true}$ and $\mathsf{false}$. Most propositions we consider have a corresponding Boolean-valued decision function. In Coq we use type classes to keep track of these correspondences, but in this paper we leave these correspondences implicit.

### **Definition 2.3**

We let $\mathsf{option}\;A$ denote the *option type over* *A*, whose elements are inductively defined as either $\bot$ or $x$ for some $x \in A$. We implicitly lift operations to operate on the option type, and often omit cases of definitions that yield $\bot$. This is formally described using the *option monad* in the Coq formalization.

### **Definition 2.4**

A *partial function* *f* *from* *A* *to* *B* is a function $f : A \rightarrow \mathsf{option}\;B$.

### **Definition 2.5**

A partial function *f* is called *a finite partial function* or a *finite map* if its *domain* $\mathsf{dom}\;f :=\{ x \;|\;\exists y\in B\,.\,f\,x = y\}$ is finite. The type of finite partial functions is denoted as $A \rightarrow _{\mathsf{fin}} B$. The operation ${f}[ {x} := {y} ]$ yields *f* with the value *y* for argument *x*.

### **Definition 2.6**

We let $A \times B$ denote *the product of types* *A* *and* *B*. Given a *pair* $(x,\,y) \in A \times B$, we let ${(x,\,y)}_{\mathbf {1}} :=x$ and ${(x,\,y)}_{\mathbf {2}} :=y$ denote the *first* and *second projection* of $(x,\,y)$.

### **Definition 2.7**

We let $\mathsf{list}\;A$ denote the *list type over* *A*, whose elements are inductively defined as either $\varepsilon$ or $x\,{x}$ for some $x \in A$ and ${{x}} \in \mathsf{list}\;A$. We let $x_i \in A$ denote the *i*th element of a list ${{x}} \in \mathsf{list}\;A$ (we count from 0). Lists are sometimes denoted as $[\, x_0,\ldots ,x_{n-1} \,] \in \mathsf{list}\;A$ for $x_0,\ldots ,x_{n-1} \in A$.

We use the following operations on lists:

- We often implicitly lift a function $f : A_0 \rightarrow \cdots \rightarrow A_n$ point-wise to the function $f : \mathsf{list}\;A_0 \rightarrow \cdots \rightarrow \mathsf{list}\;A_n$. The resulting list is truncated to the length of the smallest input list in case $n > 1$.
- We often implicitly lift a predicate $P : A_0 \rightarrow A_{n-1} \rightarrow \mathsf{Prop}$ to the predicate $P : \mathsf{list}\;A_0 \rightarrow \cdots \rightarrow \mathsf{list}\;A_{n-1} \rightarrow \mathsf{Prop}$ that guarantees that *P* holds for all (pairs of) elements of the list(s). The lifted predicate requires all lists to have the same length in case $n > 1$.
- We let $|{{x}}| \in \mathbb {N}$ denote the length of ${{x}} \in \mathsf{list}\;A$.
- We let ${{x}}_{[i,\,j)} \in \mathsf{list}\;A$ denote the sublist $x_i \ldots x_{j-1}$ of ${{x}} \in \mathsf{list}\;A$.
- We let $x ^ {n} \in \mathsf{list}\;A$ denote the list consisting of *n* times $x \in A$.
- We let ${({{{x}}} {y}^\infty )}_{[i,\,j)} \in \mathsf{list}\;A$ denote the sublist $x_i \ldots x_{j-1}$ of ${{x}} \in \mathsf{list}\;A$ which is padded with $y \in A$ in case ${{x}}$ is too short.
- Given lists ${{x}} \in \mathsf{list}\;A$ and ${{y}} \in \mathsf{list}\;B$ with $|{{x}}| = |{{y}}|$, we let  denote the point-wise pairing of ${{x}}$ and ${{y}}$.

## Challenges

This section illustrates a number of subtle forms of underspecification in C by means of example programs, their bizarre behaviors exhibited by widely used C compilers, and their treatment in $\mathrm{CH}_2\mathrm{O}$. Many of these examples involve delicacies due to the interaction between the following two ways of accessing data:

- In a *high-level* way using arrays, structs and unions.
- In a *low-level* way using unstructured and untyped byte representations.

The main problem is that compilers use a high-level view of data access to perform optimizations whereas both programmers and traditional memory models expect data access to behave in a concrete low-level way.

### Byte-Level Operations and Object Representations

Apart from *high-level* access to objects in memory by means of typed expressions, C also allows *low-level* access by means of byte-wise manipulation. Each object of type $\tau$ can be interpreted as an unsigned char array of length $\mathtt {sizeof(\tau )}$, which is called the *object representation* [27, 6.2.6.1p4]. Let us consider:
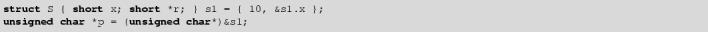


On 32-bit computing architectures such as x86 (with _Alignof(short*) $=4$), the object representation of s1 might be:


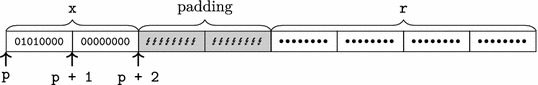


The above object representation contains a hole due to *alignment* of objects. The bytes belonging to such holes are called *padding bytes*.

Alignment is the way objects are arranged in memory. In modern computing architectures, accesses to addresses that are a multiple of a word sized chunk (often a multiple of 4 bytes on a 32-bit computing architecture) are significantly faster due to the way the processor interacts with the memory. For that reason, the C11 standard has put restrictions on the addresses at which objects may be allocated [27, 6.2.8]. For each type $\tau$, there is an implementation-defined integer constant _Alignof($\tau$), and objects of type $\tau$ are required to be allocated at addresses that are a multiple of that constant. In case _Alignof(short*) $=4$, there are thus two bytes of padding in between the fields of struct S.

An object can be copied by copying its object representation. For example, the struct s1 can be copied to s2 as follows:
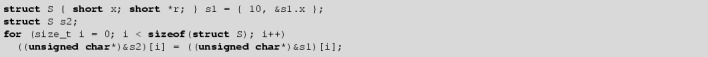


In the above code, size_t is an unsigned integer type, which is able to hold the results of the sizeof operator [27, 7.19p2].

Manipulation of object representations of structs also involves access to padding bytes, which are not part of the high-level representation. In particular, in the example the padding bytes are also being copied. The problematic part is that padding bytes have indeterminate values, whereas in general, reading an indeterminate value has undefined behavior (for example, reading from an uninitialized int variable is undefined). The C11 standard provides an exception for unsigned char [27, 6.2.6.1p5], and the above example thus has defined behavior.

Our memory model uses a symbolic representation of bits (Definition 6.19) to distinguish determinate and indeterminate memory. This way, we can precisely keep track of the situations in which access to indeterminate memory is permitted.

### Padding of Structs and Unions

The following excerpt from the C11 standard points out another challenge with respect to padding bytes [27, 6.2.6.1p6]:

When a value is stored in an object of structure or union type, including in a member object, the bytes of the object representation that correspond to any padding bytes take unspecified values.

Let us illustrate this difficulty by an example:
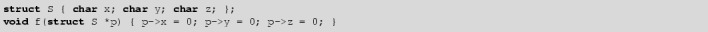


On architectures with sizeof(struct S) = 4, objects of type struct S have one byte of padding. The object representation may be as follows:


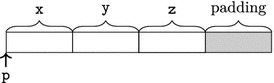


Instead of compiling the function f to three store instructions for each field of the struct, the C11 standard allows a compiler to use a single instruction to store zeros to the entire struct. This will of course affect the padding byte. Consider:
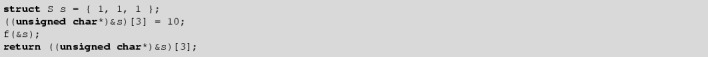


Now, the assignments to fields of s by the function f affect also the padding bytes of s, including the one ((unsigned char*)&s)[3] that we have assigned to. As a consequence, the returned value is unspecified.

From a high-level perspective this behavior makes sense. Padding bytes are not part of the abstract value of a struct, so their actual value should not matter. However, from a low-level perspective it is peculiar. An assignment to a specific field of a struct affects the object representation of parts not assigned to.

None of the currently existing C formalizations describes this behavior correctly. In our tree based memory model we enforce that padding bytes always have an indeterminate value, and in turn we have the desired behavior implicitly. Note that if the function call f(&s) would have been removed, the behavior of the example program remains unchanged in $\mathrm{CH}_2\mathrm{O}$.

### Type-Punning

Despite the rules for effective types, it is under certain conditions nonetheless allowed to access a union through another variant than the current one. Accessing a union through another variant is called *type-punning*. For example:
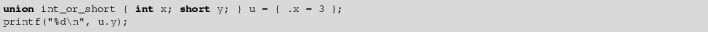


This code will reinterpret the bit representation of the int value 3 of u.x as a value of type short. The reinterpreted value that is printed is implementation-defined (on architectures where shorts do not have trap values).

Since C11 is ambiguous about the exact conditions under which type-punning is allowed,^1^ we follow the interpretation by the GCC documentation [20]:

Type-punning is allowed, provided the memory is accessed through the union type.

According to this interpretation the above program indeed has implementation defined behavior because the variant y is accessed via the expression u.y that involves the variable u of the corresponding union type.

However, according to this interpretation, type-punning via a pointer to a specific variant of a union type yields undefined behavior. This is in agreement with the rules for effective types. For example, the following program has undefined behavior.
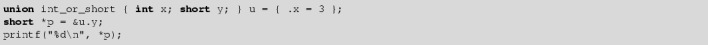


We formalize the interpretation of C11 by GCC by decorating pointers and l-values to subobjects with annotations (Definition 6.4). When a pointer to a variant of a union is stored in memory, or used as the argument of a function, the annotations are changed to ensure that type-punning no longer has defined behavior via that pointer. In Sect. 7.1 we formally establish that this approach is correct by showing that a compiler can perform type-based alias analysis (Theorem 7.2 on p. 51).

### Indeterminate Memory and Pointers

A pointer value becomes indeterminate when the object it points to has reached the end of its lifetime [27, 6.2.4] (it has gone out of scope, or has been deallocated). Dereferencing an indeterminate pointer has of course undefined behavior because it no longer points to an actual value. However, not many people are aware that using an indeterminate pointer in pointer arithmetic and pointer comparisons also yields undefined behavior. Consider:
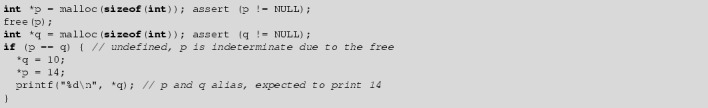


In this code malloc(sizeof(int)) yields a pointer to a newly allocated memory area that may hold an integer, or yields a NULL pointer in case no memory is available. The function free deallocates memory allocated by malloc. In the example we assert that both calls to malloc succeed.

After execution of the second call to malloc it may happen that the memory area of the first call to malloc is reused: we have used free to deallocate it after all. This would lead to the following situation in memory:


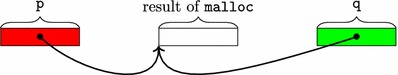


Both GCC (version 4.9.2) or Clang (version 3.5.0) use the fact that p and q are obtained via different calls to malloc as a license to assume that p and q do not alias. As a result, the value 10 of *q is inlined, and the program prints the value 10 instead of the naively expected value 14.

The situation becomes more subtle because when the object a pointer points to has been deallocated, not just the argument of free becomes indeterminate, but also all other copies of that pointer. This is therefore yet another example where high-level representations interact subtly with their low-level counterparts.

In our memory model we represent pointer values symbolically (Definition 6.4), and keep track of memory areas that have been previously deallocated. The behavior of operations like == depends on the memory state, which allows us to accurately capture the described undefined behaviors.

### End-of-Array Pointers

The way the C11 standard deals with pointer equality is subtle. Consider the following excerpt [27, 6.5.9p6]:

Two pointers compare equal if and only if [...] or one is a pointer to one past the end of one array object and the other is a pointer to the start of a different array object that happens to immediately follow the first array object in the address space.

End-of-array pointers are peculiar because they cannot be dereferenced, they do not point to any value after all. Nonetheless, end-of-array are commonly used when looping through arrays.
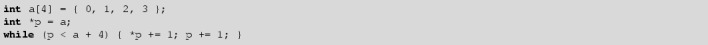


The pointer p initially refers to the first element of the array a. The value p points to, as well as p itself, is being increased as long as p is before the end-of-array pointer a + 4. This code thus increases the values of the array a. The initial state of the memory is displayed below:


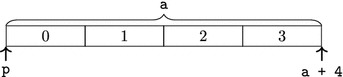


End-of-array pointers can also be used in a way where the result of a comparison is not well-defined. In the example below, the printf is executed only if x and y are allocated adjacently in the address space (typically the stack).
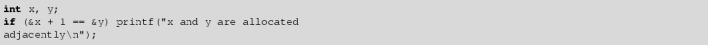


Based on the aforementioned excerpt of the C11 standard [27, 6.5.9p6], one would naively say that the value of &x + 1 ==&y is uniquely determined by the way x and y are allocated in the address space. However, the GCC implementers disagree.^2^ They claim that Defect Report #260 [26] allows them to take the *derivation of a pointer value* into account.

In the example, the pointers &x + 1 and &y are derived from unrelated objects (the local variables x and y). As a result, the GCC developers claim that &x + 1 and &y may compare unequal albeit being allocated adjacently. Consider:
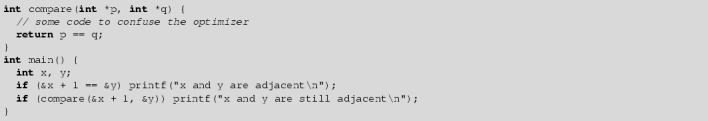


When compiled with GCC (version 4.9.2), we have observed that the string x and y are still adjacent is being printed, wheras x and y are adjacent is not being printed. This means that the value of &x + 1 ==&y is not consistent among different occurrences of the comparison.

Due to these discrepancies we assign undefined behavior to questionable uses of end-of-array pointers while assigning the correct defined behavior to pointer comparisons involving end-of-array pointers when looping through arrays (such as in the first example above). Our treatment is similar to our extension of CompCert [34].

### Sequence Point Violations and Non-determinism

Instead of having to follow a specific execution order, the execution order of expressions is unspecified in C. This is a common cause of portability problems because a compiler may use an arbitrary execution order for each expression, and each time that expression is executed. Hence, to ensure correctness of a C program with respect to an arbitrary compiler, one has to verify that *each* possible execution order is free of undefined behavior and gives the correct result.

In order to make more effective optimizations possible (for example, delaying of side-effects and interleaving), the C standard does not allow an object to be modified more than once during the execution of an expression. If an object is modified more than once, the program has undefined behavior. We call this requirement the *sequence point restriction*. Note that this is not a static restriction, but a restriction on valid executions of the program. Let us consider an example:
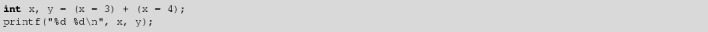


By considering all possible execution orders, one would naively expect this program to print 4 7 or 3 7, depending on whether the assignment x = 3 or x = 4 is executed first. However, x is modified twice within the same expression, and thus both execution orders have undefined behavior. The program is thereby allowed to exhibit any behavior. Indeed, when compiled with gcc -O2 (version 4.9.2), the compiled program prints 4 8, which does not correspond to any of the execution orders.

Our approach to non-determinism and sequence points is inspired by Norrish [44] and Ellison and Roşu [19]. Each bit in memory carries a permission (Definition 5.5) that is set to a special *locked* permission when a store has been performed. The memory model prohibits any access (read or store) to objects with locked permissions. At the next sequence point, the permissions of locked objects are changed back into their original permission, making future accesses possible again.

It is important to note that we do not have non-determinism in the memory model itself, and have set up the memory model in such a way that all non-determinism is on the level of the small-step operational semantics.

## Types in C

This section describes the types used in the $\mathrm{CH}_2\mathrm{O}$ memory model. We support integer, pointer, function pointer, array, struct, union and void types. More complicated types such as enum types and typedefs are defined by translation [33, 37].

This section furthermore describes an abstract interface, called an *implementation environment*, that describes properties such as size and endianness of integers, and the layout of structs and unions. The entire $\mathrm{CH}_2\mathrm{O}$ memory model and semantics will be parameterized by an implementation environment.

### Integer Representations

This section describes the part of implementation environments corresponding to integer types and the encoding of integer values as bits. Integer types consist of a *rank* ($\mathsf{char}$, $\mathsf{short}$, $\mathsf{int}$ ...) and a *signedness* ($\mathsf{signed}$ or $\mathsf{unsigned}$). The set of available ranks as well as many of their properties are implementation-defined. We therefore abstract over the ranks in the definition of integer types.

#### **Definition 4.1**

*Integer signedness* and *integer types* over ranks $k \in K$ are inductively defined as:The projections are called $\mathsf{rank}: \mathsf{inttype}\rightarrow K$ and $\mathsf{sign}: \mathsf{inttype}\rightarrow \mathsf{signedness}$.

#### **Definition 4.2**

An *integer coding environment with ranks* *K* consists of a total order $(K,\subseteq )$ of *integer ranks* having at least the following ranks:

$$\begin{aligned} \mathsf{char}\subset \mathsf{short}\subset \mathsf{int}\subset \mathsf{long}\subset \mathsf{long}\;\mathsf{long}\qquad \text {and}\qquad \mathsf{ptr\_rank}. \end{aligned}$$

It moreover has the following functions:Here, $\mathsf{endianize}\;k$ and $\mathsf{deendianize}\;k$ should be inverses, $\mathsf{endianize}\;k$ should be a permutation, $\mathsf{rank\_size}$ should be (non-strictly) monotone, and $\mathsf{rank\_size}\;\mathsf{char}= 1$.

#### **Definition 4.3**

The judgment $x : {\tau }_{\mathsf{i}}$ describes that $x \in \mathbb {Z}$ *has integer type* ${\tau }_{\mathsf{i}}$.


The rank $\mathsf{char}$ is the rank of the smallest integer type, whose unsigned variant corresponds to bytes that constitute object representations (see Sect. 3.1). Its bit size is $\mathsf{char\_bits}$ (called CHAR_BIT in the standard library header files [27, 5.2.4.2.1]), and its signedness $\mathsf{char\_signedness}$ is implementation-defined [27, 6.2.5p15].

The rank $\mathsf{ptr\_rank}$ is the rank of the integer types size_t and ptrdiff_t, which are defined in the standard library header files [27, 7.19p2]. The type ptrdiff_t is a signed integer type used to represent the result of subtracting two pointers, and the type size_t is an unsigned integer type used to represent sizes of types.

An integer coding environment can have an arbitrary number of integer ranks apart from the standard ones $\mathsf{char}$, $\mathsf{short}$, $\mathsf{int}$, $\mathsf{long}$, $\mathsf{long}\;\mathsf{long}$, and $\mathsf{ptr\_rank}$. This way, additional integer types like those describe in [27, 7.20] can easily be included.

The function $\mathsf{rank\_size}$ gives the byte size of an integer of a given rank. Since we require $\mathsf{rank\_size}$ to be monotone rather than strictly monotone, integer types with different ranks can have the same size [27, 6.3.1.1p1]. For example, on many implementations int and long have the same size, but are in fact different.

The C11 standard allows implementations to use either sign-magnitude, 1’s complement or 2’s complement signed integers representations. It moreover allows integer representations to contain padding or parity bits [27, 6.2.6.2]. However, since all current machine architectures use 2’s complement representations, this is more of a historic artifact. Current machine architectures use 2’s complement representations because these do not suffer from positive and negative zeros and thus enjoy unique representations of the same integer. Hence, $\mathrm{CH}_2\mathrm{O}$ restricts itself to implementations that use 2’s complement signed integers representations.

Integer representations in $\mathrm{CH}_2\mathrm{O}$ can solely differ with respect to endianness (the order of the bits). The function $\mathsf{endianize}$ takes a list of bits in little endian order and permutes them accordingly. We allow $\mathsf{endianize}$ to yield an arbitrary permutation and thus we not just support big- and little-endian, but also mixed-endian variants.

#### **Definition 4.4**

Given an integer type ${\tau }_{\mathsf{i}}$, the *integer encoding functions*  and $(\_)_{{\tau }_{\mathsf{i}}} : \mathsf{list}\;\mathsf{bool}\rightarrow \mathbb {Z}$ are defined as follows:


#### **Lemma 4.5**

The integer encoding functions are inverses. That means:

1. We have  and  provided that $x : {\tau }_{\mathsf{i}}$.
2. We have  and $({\beta })_{{\tau }_{\mathsf{i}}} : {\tau }_{\mathsf{i}}$ provided that $|{\beta }| = \mathsf{rank\_size}\;{\tau }_{\mathsf{i}}$.

### Definition of Types

We support integer, pointer, function pointer, array, struct, union and void types. The translation that we have described in [33, 37] translates more complicated types, such as typedefs and enums, into these simplified types. This translation also alleviates other simplifications of our simplified definition of types, such as the use of unnamed struct and union fields. Floating point types and type qualifiers like const and volatile are not supported.

All definitions in this section are implicitly parameterized by an integer coding environment with ranks *K* (Definition 4.2).

#### **Definition 4.6**

*Tags* $t \in \mathsf{tag}$ (sometimes called *struct/union names*) and *function names* $f \in \mathsf{funname}$ are represented as strings.

#### **Definition 4.7**

*Types* consist of *point-to types*, *base types* and *full types*. These are inductively defined as:


The three mutual inductive parts of types correspond to the different components of the memory model. Addresses and pointers have point-to types (Definitions 6.8 and 6.10), base values have base types (Definition 6.40), and memory trees and values have full types (Definitions 6.25 and 6.46).

The void type of C is used for two entirely unrelated purposes: void is used for functions without return type and void* is used for pointers to objects of unspecified type. In $\mathrm{CH}_2\mathrm{O}$ this distinction is explicit in the syntax of types. The type $\mathsf{void}$ is used for function without return value. Like the mathematical *unit* type it has one value called $\mathsf{nothing}$ (Definition 6.39). The type $\mathsf{any}{*}$ is used for pointers to objects of unspecified type.

Unlike more modern programming languages C does not provide first class functions. Instead, C provides function pointers which are just addresses of executable code in memory instead of closures. Function pointers can be used in a way similar to ordinary pointers: they can be used as arguments and return value of functions, they can be part of structs, unions and arrays, *etc.*

The C language sometimes allows function types to be used as shorthands for function pointers, for example:
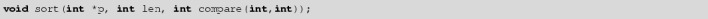


The third argument of sort is a shorthand for int (*compare)(int,int) and is thus in fact a function pointer instead of a function. We only have function pointer types, and the third argument of the type of the function sort thus contains an additional ${*}$:Struct and union types consist of just a name, and do not contain the types of their fields. An environment is used to assign fields to structs and unions, and to assign argument and return types to function names.

#### **Definition 4.8**

*Type environments* are defined as:The functions $\mathsf{dom}_\mathsf{tag}\,: \mathsf{env}\rightarrow \mathcal {P}_{\mathsf{fin}}(\mathsf{tag})$ and $\mathsf{dom}_\mathsf{funname}: \mathsf{env}\rightarrow \mathcal {P}_{\mathsf{fin}}(\mathsf{funname})$ yield the declared structs and unions, respectively the declared functions. We implicitly treat environments as functions $\mathsf{tag} \rightarrow _{\mathsf{fin}} \mathsf{list}\;\mathsf{type}$ and $\mathsf{funname} \rightarrow _{\mathsf{fin}} (\mathsf{list}\;\mathsf{type}\times \mathsf{type})$ that correspond to underlying finite partial functions.

Struct and union names on the one hand, and function names on the other, have their own name space in accordance with the C11 standard [27, 6.2.3p1].

#### **Notation 4.9**

We often write an environment as a mixed sequence of struct and union declarations $t : {\tau }$, and function declarations $f : ({\tau },\,\tau )$. This is possible because environments are finite.

Since we represent the fields of structs and unions as lists, fields are nameless. For example, the C type struct S1 { int x; struct S1 *p; } is translated into the environment $\mathtt {S1} : [\, {{\mathsf{signed}\;\mathsf{int}}}, {{\mathsf{struct}\;\mathtt {S1}}{*}} \,]$.

Although structs and unions are semantically very different (products versus sums, respectively), environments do not keep track of whether a tag has been used for a struct or a union type. Structs and union types with the same tag are thus allowed. The translator in [33, 37] forbids the same name being used to declare both a struct and union type.

Although our mutual inductive syntax of types already forbids many incorrect types such as functions returning functions (instead of function pointers), still some ill-formed types such as int[0] are syntactically valid. Also, we have to ensure that cyclic structs and unions are only allowed when the recursive definition is guarded through pointers. Guardedness by pointers ensures that the sizes of types are finite and statically known. Consider the following types:
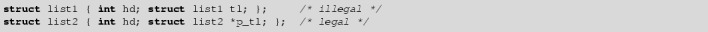


The type declaration struct list1 is illegal because it has a reference to itself. In the type declaration struct list2 the self reference is guarded through a pointer type, and therefore legal. Of course, this generalizes to mutual recursive types like:
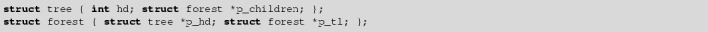


#### **Definition 4.10**

The following judgments are defined by mutual induction:

- The judgment $\Gamma \vdash _{*} {{\tau }_{\mathsf{p}}}$ describes *point-to types* ${\tau }_{\mathsf{p}}$ *to which a pointer may point*:
- The judgment $\Gamma \vdash _{\mathsf{b}} {{\tau }_{\mathsf{b}}}$ describes *valid base types* ${\tau }_{\mathsf{b}}$:
- The judgment $\Gamma \vdash {\tau }$ describes *valid types* $\tau$:

#### **Definition 4.11**

The judgment $\vdash \Gamma$ describes *well-formed environments* $\Gamma$. It is inductively defined as:


Note that $\Gamma \vdash {\tau }$ does not imply $\vdash \Gamma$. Most results therefore have $\vdash \Gamma$ as a premise. These premises are left implicit in this paper.

In order to support (mutually) recursive struct and union types, pointers to incomplete struct and union types are permitted in the judgment $\Gamma \vdash _{*} {{\tau }_{\mathsf{p}}}$ that describes types to which pointers are allowed, but forbidden in the judgment $\Gamma \vdash {\tau }$ of validity of types. Let us consider the following type declarations:
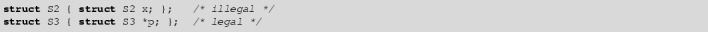


Well-formedness $\vdash \Gamma$ of the environment $\Gamma :=\mathtt {S3} : [\, {{\mathsf{struct}\;\mathtt {S3}}{*}} \,]$ can be derived using the judgments $\emptyset \vdash _{*} {\mathsf{struct}\;\mathtt {S3}}$, $\emptyset \vdash _{\mathsf{b}} {{\mathsf{struct}\;\mathtt {S3}}{*}}$, $\emptyset \vdash {{{\mathsf{struct}\;\mathtt {S3}}{*}}}$, and thus $\vdash \Gamma$. The environment $\mathtt {S2} : [\, \mathsf{struct}\;\mathtt {S2} \,]$ is ill-formed because we do not have $\emptyset \vdash {\mathsf{struct}\;\mathtt {S2}}$.

The typing rule for function pointers types is slightly more delicate. This is best illustrated by an example:
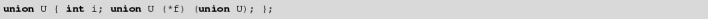


This example displays a recursive self reference to a union type through a function type, which is legal in C because function types are in fact pointer types. Due to this reason, the premises of $\Gamma \vdash _{*} {{{\tau } \rightarrow \tau }}$ are $\Gamma \vdash _{*} {{\tau }}$ and $\Gamma \vdash _{*} {\tau }$ instead of $\Gamma \vdash {{\tau }}$ and $\Gamma \vdash {\tau }$. Well-formedness of the above union type can be derived as follows:In order to define operations by recursion over the structure of well-formed types (see for example Definition 6.45, which turns a sequence of bits into a value), we often need to perform recursive calls on the types of fields of structs and unions. In Coq we have defined a custom recursor and induction principle using well-founded recursion. In this paper, we will use these implicitly.

Affeldt et al. [1, 2] have formalized non-cyclicity of types using a complex constraint on paths through types. Our definition of validity of environments (Definition 4.11) follows the structure of type environments, and is therefore well-suited to implement the aforementioned recursor and induction principle.

There is a close correspondence between array and pointer types in C. Arrays are not first class types, and except for special cases such as initialization, manipulation of arrays is achieved via pointers. We consider arrays as first class types so as to avoid having to make exceptions for the case of arrays all the time.

Due to this reason, more types are valid in $\mathrm{CH}_2\mathrm{O}$ than in C11. The translator in [33, 37] resolves exceptional cases for arrays. For example, a function parameter of array type acts like a parameter of pointer type in C11 [27, 6.7.6.3].^3^ 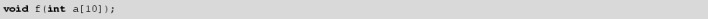


The corresponding type of the function f is thus ${{({{{\mathsf{signed}\;\mathsf{int}}}}){*}} \rightarrow {\mathsf{void}}}$. Note that the type ${{({{{\mathsf{signed}\;\mathsf{int}}}})}[10] \rightarrow {\mathsf{void}}}$ is also valid, but entirely different, and never generated by the translator in [33, 37].

### Implementation Environments

We finish this section by extending integer coding environments to describe implementation-defined properties related the layout of struct and union types. The author’s PhD thesis [33] also considers the implementation-defined behavior of integer operations (such as addition and division) and defines inhabitants of this interface corresponding to actual computing architectures.

#### **Definition 4.12**

A *implementation environment with ranks* *K* consists of an integer coding environment with ranks *K* and functions:These functions should satisfy:Here, we let $\mathsf{offsetof}_{\Gamma }\,{\tau }\;i$ denote $\Sigma _{j < i} (\mathsf{fieldsizes}_{\Gamma }\;{\tau })_j$. The functions $\mathsf{sizeof}_{\Gamma }$, $\mathsf{alignof}_{\Gamma }$, and $\mathsf{fieldsizes}_{\Gamma }$ should be closed under weakening of $\Gamma$.

#### **Notation 4.13**

Given an implementation environment, we let:


We let $\mathsf{sizeof}_{\Gamma }\;\tau$ specify the number of bytes out of which the object representation of an object of type $\tau$ consists. Objects of type $\tau$ should be allocated at addresses that are a multiple of $\mathsf{alignof}_{\Gamma }\;\tau$. We will prove that our abstract notion of addresses satisfies this property (see Lemma 6.18). The functions $\mathsf{sizeof}_{\Gamma }$, $\mathsf{alignof}_{\Gamma }$ correspond to the sizeof and _Alignof operators [27, 6.5.3.4], and $\mathsf{offsetof}_{\Gamma }$ corresponds to the offsetof macro [27, 7.19p3]. The list $\mathsf{fieldsizes}_{\Gamma }\;{\tau }$ specifies the layout of a struct type with fields ${\tau }$ as follows:


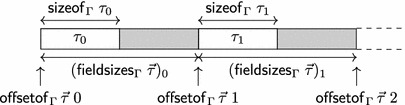


## Permissions and Separation Algebras

Permissions control whether memory operations such as a read or store are allowed or not. In order to obtain the highest level of precision, we tag each individual bit in memory with a corresponding permission. In the operational semantics, permissions have two main purposes:

- Permissions are used to formalize the *sequence point restriction* which assigns undefined behavior to programs in which an object in memory is modified more than once in between two sequence points.
- Permissions are used to distinguish objects in memory that are writable from those that are read-only (*const qualified* in C terminology).

In the axiomatic semantics based on separation logic, permissions play an important role for *share accounting*. We use share accounting for *subdivision of permissions* among multiple subexpressions to ensure that:

- Writable objects are unique to each subexpression.
- Read-only objects may be shared between subexpressions.

This distinction is originally due to Dijkstra [16] and is essential in separation logic with permissions [11]. The novelty of our work is to use separation logic with permissions for non-determinism in expressions in C. Share accounting gives rise to a natural treatment of C’s sequence point restriction.

*Separation algebras* as introduced by Calcagno et al. [13] abstractly capture common structure of subdivision of permissions. We present a generalization of separation algebras that is well-suited for C verification in Coq and use this generalization to build the permission system and memory model compositionally. The permission system will be constructed as a telescope of separation algebras:Here, $\mathbb {Q}$ is the separation algebra of fractional permissions, $\mathcal {C}$ is a functor that extends a separation algebra with a counting component, and $\mathcal {L}$ is a functor that extends a separation algebra with a lockable component (used for the sequence point restriction). This section explains these functors and their purposes.

### Separation Logic and Share Accounting

Before we will go into the details of the $\mathrm{CH}_2\mathrm{O}$ permission system, we briefly introduce separation logic. Separation logic [47] is an extension of Hoare logic that provides better means to reason about imperative programs that use mutable data structures and pointers. The key feature of separation logic is the *separating conjunction* $P \mathrel {*}Q$ that allows one to subdivide the memory into two disjoint parts: a part described by *P* and another part described by *Q*. The separating conjunction is most prominent in the *frame rule*.This rule enables local reasoning. Given a Hoare triple $\{P\}\, s\, \{Q\}$, this rule allows one to derive that the triple also holds when the memory is extended with a disjoint part described by *R*. The frame rule shows its merits when reasoning about functions. There it allows one to consider a function in the context of the memory the function actually uses, instead of having to consider the function in the context of the entire program’s memory. However, already in derivations of small programs the use of the frame rule can be demonstrated^4^:The *singleton assertion*  denotes that the memory consists of exactly one object with value *v* at address *a*. The assignments are not considered in the context of the entire memory, but just in the part of the memory that is used.

The key observation that led to our separation logic for C, see also [31, 33], is the correspondence between non-determinism in expressions and a form of concurrency. Inspired by the rule for the parallel composition [46], we have rules for each operator $\circledcirc$ that are of the following shape.The intuitive idea of this rule is that if the memory can be subdivided into two parts in which the subexpressions $e_1$ and $e_2$ can be executed safely, then the expression $e_1 \circledcirc e_2$ can be executed safely in the whole memory. Non-interference of the side-effects of $e_1$ and $e_2$ is guaranteed by the separating conjunction. It ensures that the parts of the memory described by $P_1$ and $P_2$ do not have overlapping areas that will be written to. We thus effectively rule out expressions with undefined behavior such as (x = 3) + (x = 4) (see Sect. 3.6 for discussion).

Subdividing the memory into multiple parts is not a simple operation. In order to illustrate this, let us consider a shallow embedding of assertions of separation logic $P,Q : \mathsf{mem}\rightarrow \mathsf{Prop}$ (think of $\mathsf{mem}$ as being the set of finite partial functions from some set of object identifiers to some set of objects. The exact definition in the context of $\mathrm{CH}_2\mathrm{O}$ is given in Definition 6.26). In such a shallow embedding, one would define the separating conjunction as follows:The operation  is *not* the disjoint union of finite partial functions, but a more fine grained operation. There are two reasons for that. Firstly, subdivision of memories should allow for partial overlap, as long as writable objects are unique to a single part. For example, the expression x + x has defined behavior, but the expressions x + (x = 4) and (x = 3) + (x = 4) have not.

We use separation logic with permissions [11] to deal with partial overlap of memories. That means, we equip the singleton assertion  with a permission $\gamma$. The essential property of the singleton assertion is that given a writable permission $\gamma _w$ there is a readable permission $\gamma _r$ with:The above property is an instance of a slightly more general property. We consider a binary operation  on permissions so we can write:Secondly, it should be possible to subdivide array, struct and union objects into subobjects corresponding to their elements. For example, in the case of an array int a[2], the expression (a[0] = 1) + (a[1] = 4) has defined behavior, and we should be able to prove so. The essential property of the singleton assertion for an $\mathsf{array}_{}\,{[\, y_0, \ldots , y_{n-1} \,]}$ value is:This paper does not describe the $\mathrm{CH}_2\mathrm{O}$ separation logic and its shallow embedding of assertions. These are described in the author’s PhD thesis [33]. Instead, we consider just the operations  on permissions and memories.

### Separation Algebras

As shown in the previous section, the key operation needed to define a shallow embedding of separation logic with permissions is a binary operation  on memories and permissions. Calcagno et al. introduced the notion of a *separation algebra* [13] so as to capture common properties of the  operation. A *separation algebra*  is a partial cancellative commutative monoid (see Definition 5.1 for our actual definition). Some prototypical instances of separation algebras are:

- Finite partial functions , where $\emptyset$ is the empty finite partial function, and  the disjoint union on finite partial functions.
- The Booleans $$(\mathsf{bool},\mathsf{false},\vee )$$.
- Boyland’s fractional permissions $([0,1]_\mathbb {Q},0,+)$ where 0 denotes no access, 1 denotes writable access, and $0 < \_ < 1$ denotes read-only access [11, 12].

Separation algebras are also closed under various constructs (such as products and finite functions), and complex instances can thus be built compositionally.

When formalizing separation algebras in the Coq proof assistant, we quickly ran into some problems:

- Dealing with partial operations such as  is cumbersome, see Sect. 8.3.
- Dealing with subset types (modeled as $\Sigma$-types) is inconvenient.
- Operations such as the difference operation  cannot be defined constructively from the laws of a separation algebra.

In order to deal with the issue of partiality, we turn  into a total operation. Only in case *x* and *y* are *disjoint*, notation $x\, {\mathrel {\bot }}\, y$, we require $x \,{\mathrel {\cup }}\, y$ to satisfy the laws of a separation algebra. Instead of using subsets, we equip separation algebras with a predicate $\mathsf{valid}: A \rightarrow \mathsf{Prop}$ that explicitly describes a subset of the carrier *A*. Lastly, we explicitly add a difference operation .

#### **Definition 5.1**

A *separation algebra* consists of a type *A*, with:

- An element $$\emptyset : A$$
- A predicate $$\mathsf{valid}: A \rightarrow \mathsf{Prop}$$
- Binary relations
- Binary operations

Satisfying the following laws:

1. If $\mathsf{valid}\;x$, then $\emptyset \, {\mathrel {\bot }}\, x$ and $\emptyset \,{\mathrel {\cup }}\, x = x$
2. If $x\, {\mathrel {\bot }}\, y$, then $y\, {\mathrel {\bot }}\, x$ and $x \,{\mathrel {\cup }}\, y = y \,{\mathrel {\cup }}\, x$
3. If $x\, {\mathrel {\bot }}\, y$ and $x \,{\mathrel {\cup }}\, y\, {\mathrel {\bot }}\, z$, then $y\, {\mathrel {\bot }}\, z$, $x\, {\mathrel {\bot }}\, y \,{\mathrel {\cup }}\, z$, and $x \,{\mathrel {\cup }}\, (y \,{\mathrel {\cup }}\, z) = (x \,{\mathrel {\cup }}\, y) \,{\mathrel {\cup }}\, z$
4. If $z\, {\mathrel {\bot }}\, x$, $z\, {\mathrel {\bot }}\, y$ and $z \,{\mathrel {\cup }}\, x = z \,{\mathrel {\cup }}\, y$, then $x = y$
5. If $x\, {\mathrel {\bot }}\, y$, then $\mathsf{valid}\;x$ and $\mathsf{valid}\;(x \,{\mathrel {\cup }}\, y)$
6. If $x\, {\mathrel {\bot }}\, y$ and $x \,{\mathrel {\cup }}\, y = \emptyset$, then $x = \emptyset$
7. If $$x\, {\mathrel {\bot }}\, y$$, then
8. If , then  and

Laws 1–4 describe the traditional laws of a separation algebra: identity, commutativity, associativity and cancellativity. Law 5 ensures that $\mathsf{valid}$ is closed under the  operation. Law 6 describes positivity. Laws 7 and 8 fully axiomatize the  relation and  operation. Using the positivity and cancellation law, we obtain that  is a partial order in which  is order preserving and respecting.

In case of permissions, the $\emptyset$ element is used to split objects of compound types (arrays and structs) into multiple parts. We thus use separation algebras instead of *permission algebras* [47], which are a variant of separation algebras without an $\emptyset$ element.

#### **Definition 5.2**

The *Boolean separation algebra* $\mathsf{bool}$ is defined as:


In the case of fractional permissions $[0,1]_\mathbb {Q}$ the problem of partiality and subset types already clearly appears. The  operation (here $+$) can ‘overflow’. We remedy this problem by having all operations operate on pre-terms (here $\mathbb {Q}$) and the predicate $\mathsf{valid}$ describes validity of pre-terms (here $0 \le \_ \le 1$).

#### **Definition 5.3**

The *fractional separation algebra* $\mathbb {Q}$ is defined as:


The version of separation algebras by Klein et al. [29] in Isabelle also models  as a total operation and uses a relation . There are some differences:

- We include a predicate $\mathsf{valid}$ to prevent having to deal with subset types.
- They have weaker premises for associativity (law 3), namely $x\, {\mathrel {\bot }}\, y$, $y\, {\mathrel {\bot }}\, z$ and $x\, {\mathrel {\bot }}\, z$ instead of $x\, {\mathrel {\bot }}\, y$ and $x \,{\mathrel {\cup }}\, y\, {\mathrel {\bot }}\, z$. Ours are more natural, *e.g.* for fractional permissions one has $0.5\, {\mathrel {\bot }}\, 0.5$ but not $0.5 + 0.5\, {\mathrel {\bot }}\, 0.5$, and it thus makes no sense to require $0.5 \,{\mathrel {\cup }}\, (0.5 \,{\mathrel {\cup }}\, 0.5) = (0.5 \,{\mathrel {\cup }}\, 0.5) \,{\mathrel {\cup }}\, 0.5$ to hold.
- Since Coq (without axioms) does not have a choice operator, the  operation cannot be defined in terms of . Isabelle has a choice operator.

Dockins et al. [17] have formalized a hierarchy of different separation algebras in Coq. They have dealt with the issue of partiality by treating  as a relation instead of a function. This is unnatural, because equational reasoning becomes impossible and one has to name all auxiliary results.

Bengtson et al. [6] have formalized separation algebras in Coq to reason about object-oriented programs. They have treated  as a partial function, and have not defined any complex permission systems.

### Permissions

In this section we define the $\mathrm{CH}_2\mathrm{O}$ permission system and show that it forms a separation algebra. We furthermore define *permission kinds*, which are used to classify the abilities of the permissions.

#### **Definition 5.4**

The lattice of *permission kinds* $(\mathsf{pkind},\subseteq )$ is defined as:


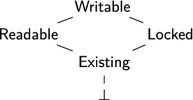


The order $k_1 \subseteq k_2$ expresses that $k_1$ has fewer abilities than $k_2$. This organization of permissions is inspired by that of Leroy et al. [40]. The intuitive meaning of the above permission kinds is as follows:

- $\mathsf{Writable}$. *Writable permissions* allow reading and writing.
- $\mathsf{Readable}$. *Read-only permissions* allow solely reading.
- $\mathsf{Existing}$. *Existence permissions* [11] are used for objects that are known to exist but whose value cannot be used. Existence permissions are used to model that C only allows pointer arithmetic on pointers that refer to objects that have not been previously deallocated (see Sect. 3.4 for discussion).
- $\mathsf{Locked}$. *Locked permissions* are used to formalize the sequence point restriction. When an object is modified during the execution of an expression, it is temporarily given a locked permission to forbid any read/write accesses until the next sequence point. For example, in (x = 3) + *p; the assignment x = 3 locks the permissions of the object x. Since future read/write accesses to x are forbidden, accessing *p results in undefined in case p points to x. At the sequence point “;”, the original permission of x is restored.
  Locked permissions are different from existence permissions because the operational semantics can change writable permissions into locked permissions and *vice versa*, but cannot do that with existence permissions.
- $\bot$. *Empty permissions* allow no operations.

In the $\mathrm{CH}_2\mathrm{O}$ separation logic we do not only have control which operations are allowed, but we also have to deal with share accounting.

- We need to subdivide objects with writable or read-only permission into multiple parts with read-only permission. For example, in the expression x + x, both subexpressions require x to have at least read-only permission.
- We need to subdivide objects with writable permission into a part with existence permission and a part with writable permission. For example, in the expression *(p + 1) = (*p = 1), the subexpression *p = 1 requires *p to have writable permission, and the subexpression *(p + 1) requires *p to have at least existence permission in order to perform pointer arithmetic on p.

We combine fractional permissions with counting permissions to combine these kinds of share accounting. Counting permissions have originally been introduced by Bornat et al. [11].

#### **Definition 5.5**

$\mathrm{CH}_2\mathrm{O}$ *permissions* $\mathsf{perm}$ are defined as:


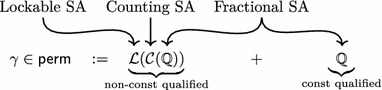


where  and $$\mathcal {C}(A) :=\mathbb {Q}\times A$$.

The author’s PhD thesis [33] gives the exact definition of the separation algebra structure of permissions by defining it one by one for the counting separation algebra $\mathcal {C}$, the lockable separation algebra $\mathcal {L}$, and the separation algebra on sums $+$. We omit the formal definitions of these separation algebras in this paper.

We have three sorts of permissions:

- Unlocked permissions  where $x \in \mathbb {Q}$ counts the number existence permissions, and $y \in \mathbb {Q}$ is a fractional permission accounting for the read/write share. Permissions  with $x < 0$ are existence permissions (see also Definitions 5.6 and 5.9). Note that the counter *x* is not a fractional permission and is thus not restricted to the interval $[0,1]_\mathbb {Q}$.
- Locked permissions  where $x \in \mathbb {Q}$ counts the number existence permissions, and $y \in \mathbb {Q}$ is a fractional permission accounting for the read/write share.
- Const permissions $\gamma \in \mathbb {Q}$, which are used for const qualified objects. Modifying an object with const permissions results in undefined behavior. Const permissions do not have a locked variant or an existence counter as they do not allow writing.

**Fig. 1:**
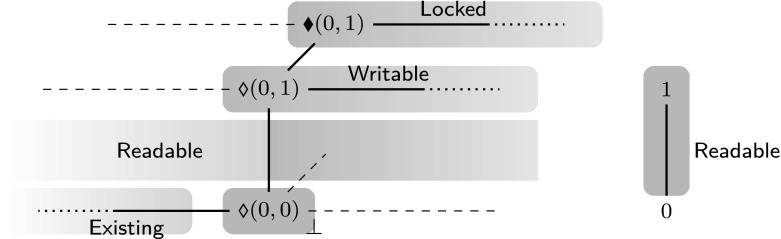
$\mathrm{CH}_2\mathrm{O}$ permissions. The *dashed lines* correspond to permissions that are invalid

The areas marked green in Fig. 1 indicate the definition of the $\mathsf{valid}$ predicate on permissions. The figure furthermore visualizes how the permissions are projected onto their kinds, which is defined formally below.

#### **Definition 5.6**

The function $\mathsf{kind}: \mathsf{perm}\rightarrow \mathsf{pkind}$ gives the *kind of a permission*. It is defined as follows:


#### **Definition 5.7**

The *locking operations* $\mathsf{lock}, \mathsf{unlock}: \mathsf{perm}\rightarrow \mathsf{perm}$ are defined as:The $\mathsf{lock}$ operation should only be used on permissions $\gamma$ with $\mathsf{Writable}\subseteq \mathsf{kind}\;\gamma$. In other cases it produces a dummy value. Likewise, $\mathsf{unlock}$ should only be used on permissions $\gamma$ with $\mathsf{kind}\;\gamma = \mathsf{Locked}$, and produces a dummy otherwise.

The operation  on permissions is defined as point-wise addition of the counting permission and the fractional permission, and the operation  is defined as point-wise subtraction. The exact definitions can be found in [33]. Apart from the common separation algebra connectives, we define an operation $\tfrac{1}{2}$ to subdivide a writable or read-only permission into two read-only permissions.

#### **Definition 5.8**

The *split operation* $\tfrac{1}{2}: \mathsf{perm}\rightarrow \mathsf{perm}$ is defined as:


Given a writable or read-only permission $\gamma$, the subdivided read-only permission $\tfrac{1}{2}\gamma$ enjoys $\tfrac{1}{2}\gamma \, {\mathrel {\bot }}\, \tfrac{1}{2}\gamma$ and $\tfrac{1}{2}\gamma \,{\mathrel {\cup }}\, \tfrac{1}{2}\gamma = \gamma$. The split operation will be described abstractly by extended separation algebras in Sect. 5.4.

#### **Definition 5.9**

The *existence permission* $\mathsf{token}$ is defined as .

Existence permissions are used to subdivide objects with writable permission into a part with existence permission and a part with writable permission. For example, in *(p + 1) = (*p = 1), the subexpression *p = 1 requires writable permission of *p, and *(p + 1) requires an existence permission of *p to perform pointer arithmetic. Subdivision is achieved using the  operation, which can be used to split a writable permission $\gamma$ into an existence permission $\mathsf{token}$ and writable permission . Law 8 of separation algebras guarantees that combining the subdivided permissions gives back the original permission, *i.e.* . Note that because $\mathsf{token}$s can be combined using  and subdivided using $\tfrac{1}{2}$, the counter *x* in the permissions  and  is an arbitrary rational number.

As ensured by Definition 6.58, only objects with the full  permission can be deallocated, whereas objects with  permission cannot. This is to model that expressions such as (p == p) + (free(p),0) have undefined behavior.

The following lemma shows that the operations on permissions interact accordingly and respect the permission kinds.

#### **Lemma 5.10**

Permissions satisfy the following properties:


### Extended Separation Algebras

We extend the notion of a separation algebra with a split operation $\tfrac{1}{2}$, and predicates $\mathsf{unmapped}$ and $\mathsf{exclusive}$ that associate – in an abstract way – an intended semantics to elements of a separation algebra. Recall that the split operation $\tfrac{1}{2}$ plays an important role in the $\mathrm{CH}_2\mathrm{O}$ separation logic [31, 33] to subdivide objects with writable or read-only permission into multiple parts with read-only permission.

#### **Definition 5.11**

An *extended separation algebra* extends a separation algebra with:

- Predicates $$\mathsf{splittable}, \mathsf{unmapped}, \mathsf{exclusive}: A \rightarrow \mathsf{Prop}$$
- A unary operation $$\tfrac{1}{2}: A \rightarrow A$$

Satisfying the following laws:

9. If $$x\, {\mathrel {\bot }}\, x$$, then $$\mathsf{splittable}\;(x \,{\mathrel {\cup }}\, x)$$
10. If $\mathsf{splittable}\;x$, then $\tfrac{1}{2}x\, {\mathrel {\bot }}\, \tfrac{1}{2}x$ and $\tfrac{1}{2}x \,{\mathrel {\cup }}\, \tfrac{1}{2}x = x$
11. If $\mathsf{splittable}\;y$ and , then $\mathsf{splittable}\;x$
12. If $x\, {\mathrel {\bot }}\, y$ and $\mathsf{splittable}\;(x \,{\mathrel {\cup }}\, y)$, then $\tfrac{1}{2}(x \,{\mathrel {\cup }}\, y) = \tfrac{1}{2}x \,{\mathrel {\cup }}\, \tfrac{1}{2}y$
13. $\mathsf{unmapped}\;\emptyset$, and if $\mathsf{unmapped}\;x$, then $\mathsf{valid}\;x$
14. If $\mathsf{unmapped}\;y$ and , then $\mathsf{unmapped}\;x$
15. If $x\, {\mathrel {\bot }}\, y$, $\mathsf{unmapped}\;x$ and $\mathsf{unmapped}\;y$, then $\mathsf{unmapped}\;(x \,{\mathrel {\cup }}\, y)$
16. $\mathsf{exclusive}\;x$ iff $\mathsf{valid}\;x$ and for all *y* with $x\, {\mathrel {\bot }}\, y$ we have $\mathsf{unmapped}\;y$
17. Not both $\mathsf{exclusive}\;x$ and $\mathsf{unmapped}\;x$
18. There exists an *x* with $\mathsf{valid}\;x$ and not $\mathsf{unmapped}\;x$

Note that $\tfrac{1}{2}$ is described by a total function whose result, $\tfrac{1}{2}x$, is only meaningful if $\mathsf{splittable}\;x$ holds. This is to account for locked permissions, which cannot be split. Law 11 ensures that splittable permissions are infinitely splittable, and law 12 ensures that $\tfrac{1}{2}$ distributes over .

The predicates $\mathsf{unmapped}$ and $\mathsf{exclusive}$ associate an intended semantics to the elements of a separation algebra in an abstract way. The predicate $\mathsf{unmapped}$ describes whether the permission allows its content to be used, as will become clear in the definition of the *tagged separation algebra* (Definition 5.13). The predicate $\mathsf{exclusive}$ is the dual of $\mathsf{unmapped}$. Let us consider the separation algebra of fractional permissions to describe the intended meaning of these predicates.

#### **Definition 5.12**

The *fractional separation algebra* $\mathbb {Q}$ is extended with:


Remember that permissions will be used to annotate each individual bit in memory. Unmapped permissions are *on the bottom*: they do not allow their bit to be used in any way. Exclusive permissions are *on the top*: they are the sole owner of a bit and can do anything to that bit without affecting disjoint bits.

Fractional permissions have exactly one unmapped element and exactly one exclusive element, but $\mathrm{CH}_2\mathrm{O}$ permissions have more structure. The elements of the $\mathrm{CH}_2\mathrm{O}$ permission system are classified as follows:
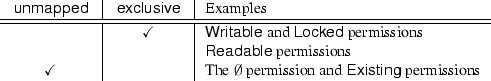


In order to formalize the intuitive meaning of the $\mathsf{unmapped}$ predicate and to abstractly describe bits annotated with permissions, we introduce the *tagged separation algebra* $\mathcal {T}_{T}^{t}(A)$. In the memory model it is instantiated as  (Definition 6.21). The elements $(\gamma ,\,b)$ consist of a permission $\gamma \in \mathsf{perm}$ and bit $b \in \mathsf{bit}$. We use the symbolic bit  that represents indeterminate storage to ensure that bits with $\mathsf{unmapped}$ permissions have no usable value.

#### **Definition 5.13**

Given a separation algebra *A* and a set of tags *T* with default tag $t \in T$, the *tagged separation algebra* $\mathcal {T}_{T}^{t}(A) :=A \times T$ *over* *A* is defined as:The definitions of the omitted relations and operations are as expected.

## The Memory Model

This section defines the $\mathrm{CH}_2\mathrm{O}$ memory model whose external interface consists of operations with the following types:


### **Notation 6.1**

We let $m \langle a \rangle _{\Gamma } :=\mathsf{lookup}_{\Gamma }\,a\;m$ and $m \langle a := v \rangle _{\Gamma } :=\mathsf{insert}_{\Gamma }\,a\;v\;m$.

Many of these operations depend on the typing environment $\Gamma$ which assigns fields to structs and unions (Definition 4.8). This dependency is required because these operations need to be aware of the layout of structs and unions.

The operation $m \langle a \rangle _{\Gamma }$ yields the value stored at address *a* in memory *m*. It fails with $\bot$ if the permissions are insufficient, effective types are violated, or *a* is an end-of-array address. Reading from (the abstract) memory is not a pure operation. Although it does not affect the memory contents, it may affect the effective types [27, 6.5p6-7]. This happens for example in case type-punning is performed (see Sect. 3.3). This impurity is factored out by the operation $\mathsf{force}_{\Gamma }\,a\;m$.

The operation $m \langle a := v \rangle _{\Gamma }$ stores the value *v* at address *a* in memory *m*. A store is only permitted in case permissions are sufficient, effective types are not violated, and *a* is not an end-of-array address. The proposition $\mathsf{writable}_{\Gamma }\,a\;m$ describes the side-conditions necessary to perform a store.

After a successful store, the operation $\mathsf{lock}_{\Gamma }\,a\;m$ is used to lock the object at address *a* in memory *m*. The lock operation temporarily reduces the permissions to $\mathsf{Locked}$ so as to prohibit future accesses to *a*. Locking yields a formal treatment of the sequence point restriction (which states that modifying an object more than once between two sequence points results in undefined behavior, see Sect. 3.6).

The operational semantics accumulates a set $\Omega \in \mathsf{lockset}$ of addresses that have been written to (Definition 6.54) and uses the operation $\mathsf{unlock}\;\Omega \;m$ at the subsequent sequence point (which may be at the semicolon that terminates a full expression). The operation $\mathsf{unlock}\;\Omega \;m$ restores the permissions of the addresses in $\Omega$ and thereby makes future accesses to the addresses in $\Omega$ possible again. The author’s PhD thesis [33] describes in detail how sequence points and locks are treated in the operational semantics.

The operation $\mathsf{alloc}_{\Gamma }\,o\;v\;\mu \;m$ allocates a new object with value *v* in memory *m*. The object has object identifier $o \notin \mathsf{dom}\;m$ which is non-deterministically chosen by the operation semantics. The Boolean $\mu$ expresses whether the new object is allocated by malloc.

Accompanying $\mathsf{alloc}_{\Gamma }$, the operation $\mathsf{free}\;o\;m$ deallocates a previously allocated object with object identifier *o* in memory *m*. In order to deallocate dynamically obtained memory via free, the side-condition $\mathsf{freeable}\;a\;m$ describes that the permissions are sufficient for deallocation, and that *a* points to a malloced object.

### Representation of Pointers

Adapted from CompCert [40, 41], we represent memory states as finite partial functions from *object identifiers* to *objects*. Each local, global and static variable, as well as each invocation of malloc, is associated with a unique object identifier of a separate object in memory. This approach separates unrelated objects by construction, and is therefore well-suited for reasoning about memory transformations.

We improve on CompCert by modeling objects as structured trees instead of arrays of bytes to keep track of padding bytes and the variants of unions. This is needed to faithfully describe C11’s notion of effective types (see page 4 of Sect. 1 for an informal description). This approach allows us to describe various undefined behaviors of C11 that have not been considered by others (see Sects. 3.1 and 3.3).

In the CompCert memory model, pointers are represented as pairs (*o*, *i*) where *o* is an object identifier and *i* is a byte offset into the object with object identifier *o*. Since we represent objects as trees instead of as arrays of bytes, we represent pointers as paths through these trees rather than as byte offsets.

#### **Definition 6.2**

*Object identifiers* $o \in \mathsf{index}$ are elements of a fixed countable set. In the Coq development we use binary natural numbers, but since we do not rely on any properties apart from countability, we keep the representation opaque.

We first introduce a typing environment to relate the shape of paths representing pointers to the types of objects in memory.

#### **Definition 6.3**

*Memory typing environments* $\Delta \in \mathsf{memenv}$ are finite partial functions $\mathsf{index} \rightarrow _{\mathsf{fin}} (\mathsf{type}\times \mathsf{bool})$. Given a memory environment $\Delta$:

1. An *object identifier* *o* *has type* $\tau$, notation $\Delta \vdash o : \tau$, if ${\Delta }\,o= (\tau ,\,\beta )$ for a $\beta$.
2. An *object identifier* *o* *is alive*, notation $\Delta \vdash o\ \mathsf{alive}$, if ${\Delta }\,o= (\tau ,\,\mathsf{false})$ for a $\tau$.

Memory typing environments evolve during program execution. The code below is annotated with the corresponding memory environments in red.
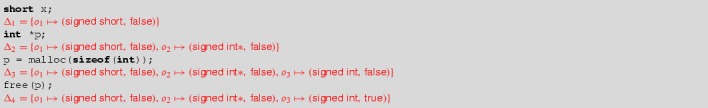


Here, $o_1$ is the object identifier of the variable x, $o_2$ is the object identifier of the variable p and $o_3$ is the object identifier of the storage obtained via malloc.

Memory typing environments also keep track of objects that have been deallocated. Although one cannot directly create a pointer to a deallocated object, existing pointers to such objects remain in memory after deallocation (see the pointer p in the above example). These pointers, also called *dangling* pointers, cannot actually be used.

#### **Definition 6.4**

*References*, *addresses* and *pointers* are inductively defined as:


References are paths from the top of an object in memory to some subtree of that object. The shape of references matches the structure of types:

- The reference  is used to select the *i*th element of a $\tau$-array of length *n*.
- The reference  is used to select the *i*th field of a struct *t*.
- The reference  is used to select the *i*th variant of a union *t*.

References can describe most pointers in C but cannot account for end-of-array pointers and pointers to individual bytes. We have therefore defined the richer notion of *addresses*. An address $(o : \tau , {{r}}, i)_{{\sigma } >_{\!*} {{\sigma }_{\mathsf{p}}}}$ consists of:

- An object identifier *o* with type $\tau$.
- A reference ${{r}}$ to a subobject of type $\sigma$ in the entire object of type $\tau$.
- An offset *i* to a particular byte in the subobject of type $\sigma$ (note that one cannot address individual bits in C).
- The type ${\sigma }_{\mathsf{p}}$ to which the address is cast. We use a points-to type in order to account for casts to the anonymous void* pointer, which is represented as the points-to type $\mathsf{any}$. This information is needed to define, for example, pointer arithmetic, which is sensitive to the type of the address.

In turn, pointers extend addresses with a NULL pointer $\mathsf{NULL}\;{\sigma }_{\mathsf{p}}$ for each type ${\sigma }_{\mathsf{p}}$, and function pointers $f^{{\tau } \mapsto \tau }$ which contain the name and type of a function.

Let us consider the following global variable declaration:
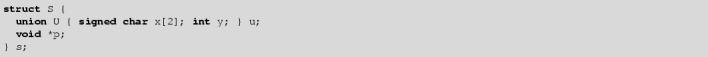


The formal representation of the pointer (void*)(s.u.x + 2) is:Here, $o_{\mathtt s}$ is the object identifier associated with the variable s of type $\mathsf{struct}\;\mathtt S$. The reference  and byte-offset 2 describe that the pointer refers to the third byte of the array s.u.x. The pointer refers to an object of type ${{\mathsf{signed}\;\mathsf{char}}}$. The annotation $\mathsf{any}$ describes that the pointer has been cast to type void*.

The annotations $q \in \{ \circ , \bullet \}$ on references  describe whether type-punning is allowed or not. The annotation $\bullet$ means that type-punning is allowed, *i.e.* accessing another variant than the current one has defined behavior. The annotation $\circ$ means that type-punning is forbidden. A pointer whose annotations are all of the shape $\circ$, and thereby does not allow type-punning at all, is called *frozen*.

#### **Definition 6.5**

The *freeze* function $|\,\_\,|_\circ : \mathsf{refseg}\rightarrow \mathsf{refseg}$ is defined as:A reference segment *r* is *frozen*, notation $\mathsf{frozen}\;r$, if $|\,r\,|_\circ = r$. Both $|\,\_\,|_\circ$ and $\mathsf{frozen}$ are lifted to references, addresses, and pointers in the expected way.

Pointers stored in memory are always in frozen shape. Definitions 6.32 and 6.41 describe the formal treatment of effective types and frozen pointers, but for now we reconsider the example from Sect. 3.3:
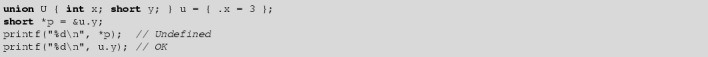


Assuming the object u has object identifier $o_{\mathtt u}$, the pointers &u.x, &u.y and p have the following formal representations:These pointers are likely to have the same object representation on actual computing architectures. However, due to effective types, &u.y may be used for type-punning but p may not. It is thus important that we distinguish these pointers in the formal memory model.

The additional structure of pointers is also needed to determine whether pointer subtraction has defined behavior. The behavior is only defined if the given pointers both point to an element of the same array object [27, 6.5.6p9]. Consider:
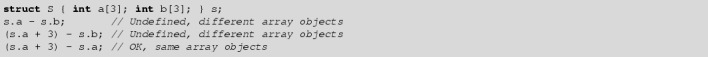


Here, the pointers s.a + 3 and s.b have different representations in the $\mathrm{CH}_2\mathrm{O}$ memory model. The author’s PhD thesis [33] gives the formal definition of pointer subtraction.

We will now define typing judgments for references, addresses and pointers. The judgment for references $\Gamma \vdash {{r}} : \tau \rightarrowtail \sigma$ states that $\sigma$ is a *subobject type of* $\tau$ which can be obtained via the reference ${{r}}$ (see also Definition 7.1). For example, int[2] is a subobject type of struct S { int x[2]; int y[3]; } via .

#### **Definition 6.6**

The judgment $\Gamma \vdash r : \tau \rightarrowtail \sigma$ describes that ${r}$ *is a valid reference from* $\tau$ *to* $\sigma$. It is inductively defined as:


The typing judgment for addresses is more involved than the judgment for references. Let us first consider the following example:
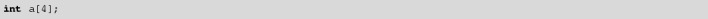


Assuming the object a has object identifier $o_{\mathtt a}$, the end-of-array pointer a+4 could be represented in at least the following ways (assuming $\mathsf{sizeof}_{}\;({{\mathsf{signed}\;\mathsf{int}}}) = 4$):In order to ensure canonicity of pointer representations, we let the typing judgment for addresses ensure that the reference ${{r}}$ of $(o : \tau , {{r}}, i)_{{\sigma } >_{\!*} {{\sigma }_{\mathsf{p}}}}$ always refers to the first element of an array subobject. This renders the second representation illegal.

#### **Definition 6.7**

The relation ${\tau } >_{\!*} {{\sigma }_{\mathsf{p}}}$, type $\tau$ *is pointer castable to* ${\sigma }_{\mathsf{p}}$, is inductively defined by ${\tau } >_{\!*} {{\tau }}$, ${\tau } >_{\!*} {{{{\mathsf{unsigned}\;\mathsf{char}}}}}$, and ${\tau } >_{\!*} {\mathsf{any}}$.

#### **Definition 6.8**

The judgment $\Gamma ,\Delta \vdash _{*} a : {\sigma }_{\mathsf{p}}$ describes that *the address* *a* *refers to type* ${\sigma }_{\mathsf{p}}$. It is inductively defined as:Here, the helper functions $\mathsf{offset}, \mathsf{size}: \mathsf{ref}\rightarrow \mathbb {N}$ are defined as:


We use an intrinsic encoding of syntax, which means that terms contain redundant type annotations so we can read off types. Functions to read off types are named $\mathsf{typeof}$ and will not be defined explicitly. Type annotations make it more convenient to define operations that depend on types (such as $\mathsf{offset}$ and $\mathsf{size}$ in Definition 6.8). As usual, typing judgments ensure that type annotations are consistent.

The premises $i \le \mathsf{sizeof}_{\Gamma }\;\sigma \cdot \mathsf{size}\;{{r}}$ and  of the typing rule ensure that the byte offset *i* is aligned and within range. The inequality $i \le \mathsf{sizeof}_{\Gamma }\;\sigma \cdot \mathsf{size}\;{{r}}$ is non-strict so as to allow end-of-array pointers.

#### **Definition 6.9**

An address $a = (o : \tau , r, i)_{{\sigma } >_{\!*} {{\sigma }_{\mathsf{p}}}}$ is called *strict*, notation $\Gamma \vdash a\ \mathsf{strict}$, in case it satisfies $i < \mathsf{sizeof}_{\Gamma }\;\sigma \cdot \mathsf{size}\;{{r}}$.

The judgment ${\tau } >_{\!*} {{\sigma }_{\mathsf{p}}}$ does not describe the typing restriction of cast expressions. Instead, it defines the invariant that each address $(o : \tau , {{r}}, i)_{{\sigma } >_{\!*} {{\sigma }_{\mathsf{p}}}}$ should satisfy. Since C is not type safe, pointer casting has ${\tau } >_{\!*} {{\sigma }_{\mathsf{p}}}$ as a run-time side-condition:
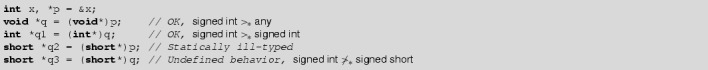


#### **Definition 6.10**

The judgment $\Gamma ,\Delta \vdash _{*} p : {\sigma }_{\mathsf{p}}$ describes that *the pointer* *p* *refers to type* ${\sigma }_{\mathsf{p}}$. It is inductively defined as:


Addresses  that point to an element of ${\tau }[n]$ always have their reference point to the first element of the array, *i.e.* $j = 0$. For some operations we use the *normalized reference* which refers to the actual array element.

#### **Definition 6.11**

The functions $\mathsf{index}: \mathsf{addr}\rightarrow \mathsf{index}$, $\mathsf{ref}_{\Gamma }: \mathsf{addr}\rightarrow \mathsf{ref}$, and $\mathsf{byte}_{\Gamma }: \mathsf{addr}\rightarrow \mathbb {N}$ obtain the *index*, *normalized reference*, and *normalized byte offset*.Here, the function $\mathsf{setoffset}: \mathbb {N}\rightarrow \mathsf{ref}\rightarrow \mathsf{ref}$ is defined as:


Let us display the above definition graphically. Given an address $(o : \tau , {{r}}, i)_{{\sigma } >_{\!*} {{\sigma }_{\mathsf{p}}}}$, the normalized reference and normalized byte offset are as follows:


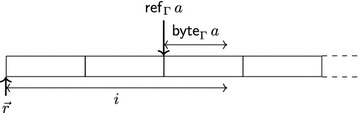


For end-of-array addresses the normalized reference is ill-typed because references cannot be end-of-array. For strict addresses the normalized reference is well-typed.

#### **Definition 6.12**

The judgment $\Delta \vdash p\ \mathsf{alive}$ describes that *the pointer* *p* *is alive*. It is inductively defined as:The judgment $\Delta \vdash o\ \mathsf{alive}$ on object identifiers is defined in Definition 6.3.

For many operations we have to distinguish addresses that refer to an entire object and addresses that refer to an individual byte of an object. We call addresses of the later kind *byte addresses*. For example:
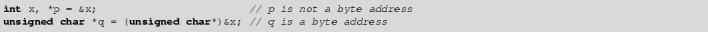


#### **Definition 6.13**

An address $(o : \tau , {{r}}, i)_{{\sigma } >_{\!*} {{\sigma }_{\mathsf{p}}}}$ is *a byte address* if ${\sigma }\ne {\sigma }_{\mathsf{p}}$.

To express that memory operations commute (see for example Lemma 6.36), we need to express that addresses are *disjoint*, meaning they do not overlap. Addresses do not overlap if they belong to different objects or take a different branch at an array or struct. Let us consider an example:
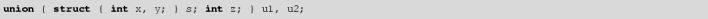


The pointers &u1 and &u2 are disjoint because they point to separate memory objects. Writing to one does not affect the value of the other and *vice versa*. Likewise, &u1.s.x and &u1.s.y are disjoint because they point to different fields of the same struct, and as such do not affect each other. The pointers &u1.s.x and &u1.z are not disjoint because they point to overlapping objects and thus do affect each other.

#### **Definition 6.14**

*Disjointness of references* ${{r}}_1$ *and* ${{r}}_2$, notation , is inductively defined as:


Note that we do not require a special case for $|\,{{r}}_1\,|_\circ \ne |\,{{r}}_2\,|_\circ$. Such a case is implicit because disjointness is defined in terms of prefixes.

#### **Definition 6.15**

*Disjointness of addresses* $a_1$ *and* $a_2$, notation , is inductively defined as:


The first inference rule accounts for addresses whose object identifiers are different, the second rule accounts for addresses whose references are disjoint, and the third rule accounts for addresses that point to different bytes of the same subobject.

#### **Definition 6.16**

The *reference bit-offset* $\mathsf{bitoffset}_{\Gamma }: \mathsf{refseg}\rightarrow \mathbb {N}$ is defined as:Moreover, we let $\mathsf{bitoffset}_{\Gamma }\,a :=\Sigma _i\, (\mathsf{bitoffset}_{\Gamma }\,(\mathsf{ref}_{\Gamma }\,a)_i) + \mathsf{byte}_{\Gamma }\,a \cdot \mathsf{char\_bits}$.

Disjointness implies non-overlapping bit-offsets, but the reverse implication does not always hold because references to different variants of unions are not disjoint. For example, given the declaration union { struct { int x, y; } s; int z; } u, the pointers corresponding to &u.s.y and &u.z are not disjoint.

#### **Lemma 6.17**

If $\Gamma ,\Delta \vdash a_1 : \sigma _1$, $\Gamma ,\Delta \vdash a_2 : \sigma _2$, $\Gamma \vdash \{a_1,a_2\}\ \mathsf{strict}$, , and $\mathsf{index}\;a_1 \ne \mathsf{index}\;a_2$, then either:

1. $$\mathsf{bitoffset}_{\Gamma }\,a_1 + \mathsf{bitsizeof}_{\Gamma }\,\sigma _1 \le \mathsf{bitoffset}_{\Gamma }\,a_2$$, or
2. $$\mathsf{bitoffset}_{\Gamma }\,a_2 + \mathsf{bitsizeof}_{\Gamma }\,\sigma _2 \le \mathsf{bitoffset}_{\Gamma }\,a_1$$.

#### **Lemma 6.18**

(Well-typed addresses are properly aligned) If $\Gamma ,\Delta \vdash a : \sigma$, then .

### Representation of Bits

As shown in Sect. 3.1, each object in C can be interpreted as an unsigned char array called the *object representation*. On actual computing architectures, the object representation consists of a sequence of concrete bits (zeros and ones). However, so as to accurately describe all undefined behaviors, we need a special treatment for the object representations of pointers and indeterminate memory in the formal memory model. To that end, $\mathrm{CH}_2\mathrm{O}$ represents the bits belonging to the object representations of pointers and indeterminate memory symbolically.

#### **Definition 6.19**

*Bits* are inductively defined as:


#### **Definition 6.20**

The judgment $\Gamma ,\Delta \vdash b$ describes that a bit *b* is *valid*. It is inductively defined as:


A bit is either a concrete bit ${0}$ or ${1}$, the *i*th fragment bit ${(\mathsf{ptr}\;{p})_{{i}}}$ of a pointer *p*, or the indeterminate bit . Integers are represented using concrete sequences of bits, and pointers as sequences of fragment bits. Assuming $\mathsf{bitsizeof}_{}\,({{{\mathsf{signed}\;\mathsf{int}}}{*}}) = 32$, a pointer *p* to a ${{\mathsf{signed}\;\mathsf{int}}}$ will be represented as the bit sequence ${(\mathsf{ptr}\;{p})_{{0}}} \ldots {(\mathsf{ptr}\;{p})_{{31}}}$, and assuming $\mathsf{bitsizeof}_{}\,({{\mathsf{signed}\;\mathsf{int}}}) = 32$ on a little-endian architecture, the integer $33 : {{\mathsf{signed}\;\mathsf{int}}}$ will be represented as the bit sequence $\mathtt {1000010000000000}$.

The approach using a combination of symbolic and concrete bits is similar to Leroy et al. [40] and has the following advantages:

- Symbolic bit representations for pointers avoid the need to clutter the memory model with subtle, implementation-defined, and run-time dependent operations to decode and encode pointers as concrete bit sequences.
- We can precisely keep track of memory areas that are uninitialized. Since these memory areas consist of arbitrary concrete bit sequences on actual machines, most operations on them have undefined behavior.
- While reasoning about program transformations one has to relate the memory states during the execution of the source program to those during the execution of the target program. Program transformations can, among other things, make more memory defined (that is, transform some indeterminate  bits into determinate bits) and relabel the memory. Symbolic bit representations make it easy to deal with such transformations (see Sect. 7.2).
- It vastly decreases the amount of non-determinism, making it possible to evaluate the memory model as part of an executable semantics [33, 37].
- The use of concrete bit representations for integers still gives a semantics to many low-level operations on integer representations.

A small difference with Leroy et al. [40] is that the granularity of our memory model is on the level of bits rather than bytes. Currently we do not make explicit use of this granularity, but it allows us to support bit-fields more faithfully with respect to the C11 standard in future work.

Objects in our memory model are annotated with permissions. We use permission annotations on the level of individual bits, rather than on the level of bytes or entire objects, to obtain the most precise way of permission handling.

#### **Definition 6.21**

*Permission annotated bits* are defined as:


In the above definition, $\mathcal {T}$ is the tagged separation algebra that has been defined in Definition 5.13. We have spelled out its definition for brevity’s sake.

#### **Definition 6.22**

The judgment $\Gamma ,\Delta \vdash \mathbf {{b}}$ describes that a permission annotated bit $\mathbf {{b}}$ is *valid*. It is inductively defined as:


### Representation of the Memory

*Memory trees* are abstract trees whose structure corresponds to the shape of data types in C. They are used to describe individual objects (base values, arrays, structs, and unions) in memory. The memory is a forest of memory trees.

#### **Definition 6.23**

*Memory trees* are inductively defined as:


The structure of memory trees is close to the structure of types (Definition 4.7) and thus reflects the expected semantics of types: arrays are lists, structs are tuples, and unions are sums. Let us consider the following example:
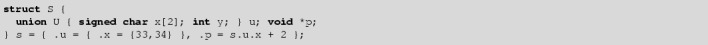


The memory tree representing the object s with object identifier $o_{\mathtt s}$ may be as follows (permissions are omitted for brevity’s sake, and integer encoding and padding are subject to implementation-defined behavior):


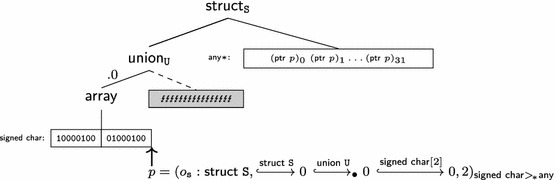


The representation of unions requires some explanation. We considered two kinds of memory trees for unions:

- The memory tree $\mathsf{union}_{t}\,(i,w,{\mathbf {{b}}})$ represents a union whose variant is *i*. Unions of variant *i* can only be accessed through a pointer to variant *i*. This is essential for effective types. The list ${\mathbf {{b}}}$ represents the padding after the element *w*.
- The memory tree $\overline{\mathsf{union}}_{t}\,{{\mathbf {{b}}}}$ represents a union whose variant is yet unspecified. Whenever the union is accessed through a pointer to variant *i*, the list ${\mathbf {{b}}}$ will be interpreted as a memory tree of the type belonging to the *i*th variant.

The reason that we consider unions $\overline{\mathsf{union}}_{t}\,{{\mathbf {{b}}}}$ with unspecific variant at all is that in some cases the variant cannot be known. Unions that have not been initialized do not have a variant yet. Also, when a union object is constructed byte-wise through its object representation, the variant cannot be known.

Although unions are tagged in the formal memory, actual compilers implement untagged unions. Information about variants should thus be internal to the formal memory model. In Sect. 7.2 we prove that this is indeed the case.

The additional structure of memory trees, namely type annotations, variants of unions, and structured information about padding, can be erased by flattening. Flattening just appends the bytes on the leaves of the tree.

#### **Definition 6.24**

The *flatten operation*  is defined as:


The flattened version of the memory tree representing the object s in the previous example is as follows:


#### **Definition 6.25**

The judgment $\Gamma ,\Delta \vdash {w} : {\tau }$ describes that the *memory tree* *w* *has type* $\tau$. It is inductively defined as:


Although padding bits should be kept indeterminate (see Sect. 3.1), padding bits are explicitly stored in memory trees for uniformity’s sake. The typing judgment ensures that the value of each padding bit is  and that the padding thus only have a permission. Storing a value in padding is a no-op (see Definition 6.35).

The side-condition $\lnot \mathsf{unmapped}\;(\overline{w}\,{{\mathbf {{b}}}})$ in the typing rule for a union $\mathsf{union}_{t}\,(i,w,{\mathbf {{b}}})$ of a specified variant ensures canonicity. Unions whose permissions are unmapped cannot be accessed and should therefore be in an unspecified variant. This condition is essential for the separation algebra structure, see Sect. 7.4.

#### **Definition 6.26**

*Memories* are defined as:


Each object $(w,\,\mu )$ in memory is annotated with a Boolean $\mu$ to describe whether it has been allocated using malloc (in case $\mu = \mathsf{true}$) or as a block scope local, static, or global variable (in case $\mu = \mathsf{false}$). The types of deallocated objects are kept to ensure that dangling pointers (which may remain to exist in memory, but cannot be used) have a unique type.

#### **Definition 6.27**

The judgment $\Gamma ,\Delta \vdash m$ describes that *the memory* *m* *is valid*. It is defined as the conjunction of:

1. For each *o* and $\tau$ with ${m}\,o = \tau$ we have:
  - $$\Delta \vdash o : \tau $$,   (b) $$\Delta \not \vdash o\ \mathsf{alive}$$, and    (c) $$\Gamma \vdash {\tau }$$.
2. For each *o*, *w* and $\mu$ with ${m}\,o = (w,\,\mu )$ we have:
  - $\Delta \vdash o : \tau$,   (b) $\Delta \vdash o\ \mathsf{alive}$,   (c) $\Gamma ,\Delta \vdash {w} : {\tau }$, and   (d) not $\overline{w}$ all .

The judgment $\Delta \vdash o\ \mathsf{alive}$ on object identifiers is defined in Definition 6.3.

#### **Definition 6.28**

The *minimal memory typing environment* $\overline{m} \in \mathsf{memenv}$ of a memory *m* is defined as:


#### **Notation 6.29**

We let $\Gamma \vdash m$ denote $\Gamma ,\overline{m} \vdash m$.

Many of the conditions of the judgment $\Gamma ,\Delta \vdash m$ ensure that the types of *m* match up with the types in the memory environment $\Delta$ (see Definition 6.3). One may of course wonder why do we not define the judgment $\Gamma \vdash m$ directly, and even consider typing of a memory in an arbitrary memory environment. Consider:
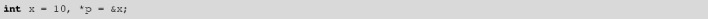


Using an assertion of separation logic we can describe the memory induced by the above program as . The separation conjunction $\mathrel {*}$ describes that the memory can be subdivided into two parts, a part for x and another part for p. When considering  in isolation, which is common in separation logic, we have a pointer that refers outside the part itself. This isolated part is thus not typeable by $\Gamma \vdash m$, but it is typeable in the context of a the memory environment corresponding to the whole memory. See also Lemma 7.26.

In the remaining part of this section we will define various auxiliary operations that will be used to define the memory operations in Sect. 6.5. We give a summary of the most important auxiliary operations:Intuitively these are just basic tree operations, but unions make their actual definitions more complicated. The indeterminate memory tree $\mathsf{new}_{\Gamma }^{\gamma }\,\tau$ consists of indeterminate bits with permission $\gamma$, the lookup operation $m [ a ]_{\Gamma }$ yields the memory tree at address *a* in *m*, and the alter operation $m[a / f]_{\Gamma }$ applies the function *f* to the memory tree at address *a* in *m*.

The main delicacy of all of these operations is that we sometimes have to interpret bits as memory trees, or reinterpret memory trees as memory trees of a different type. Most notably, reinterpretation is needed when type-punning is performed:
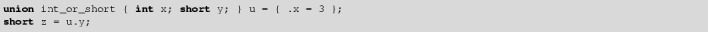


This code will reinterpret the bit representation of a memory tree representing an int value 3 as a memory tree of type short. Likewise:
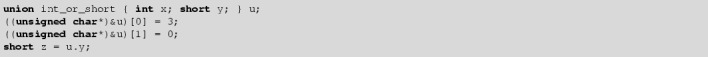


Here, we poke some bytes into the object representation of u, and interpret these as a memory tree of type short.

We have defined the flatten operation $\overline{w}$ that takes a memory tree *w* and yields its bit representation already in Definition 6.24. We now define the operation which goes in opposite direction, called the *unflatten operation*.

#### **Definition 6.30**

The *unflatten operation* $(\_)^{\tau }_{\Gamma }: \mathsf{list}\;\mathsf{pbit}\rightarrow \mathsf{mtree}$ is defined as:Here, the operation  is defined as .

Now, the need for $\overline{\mathsf{union}}_{t}\,{{{\mathbf {{b}}}}}$ memory trees becomes clear. While unflattening a bit sequence as a union, there is no way of knowing which variant of the union the bits constitute. The operations  and $(\_)^{\tau }_{\Gamma }$ are neither left nor right inverses:

- We do not have $(\overline{w})^{\tau }_{\Gamma } = w$ for each *w* with $\Gamma ,\Delta \vdash {w} : {\tau }$. Variants of unions are destroyed by flattening *w*.
- We do not have $\overline{({{\mathbf {{b}}}})^{\tau }_{\Gamma }} = {\mathbf {{b}}}$ for each ${\mathbf {{b}}}$ with $|{\mathbf {{b}}}| = \mathsf{bitsizeof}_{\Gamma }\,\tau$ either. Padding bits become indeterminate due to  by unflattening.

In Sect. 7.2 we prove weaker variants of these cancellation properties that are sufficient for proofs about program transformations.

#### **Definition 6.31**

Given a permission $\gamma \in \mathsf{perm}$, the operation $\mathsf{new}_{\Gamma }^{\gamma }: \mathsf{type}\rightarrow \mathsf{mtree}$ that yields the indeterminate memory tree is defined as:


The memory tree $\mathsf{new}_{\Gamma }^{\gamma }\,\tau$ that consists of indeterminate bits with permission $\gamma$ is used for objects with indeterminate value. We have defined $\mathsf{new}_{\Gamma }^{\gamma }\,\tau$ in terms of the unflattening operation for simplicity’s sake. This definition enjoys desirable structural properties such as $\mathsf{new}_{\Gamma }^{\gamma }\,({\tau }[n]) = (\mathsf{new}_{\Gamma }^{\gamma }\,\tau ) ^ {n}$.

We will now define the lookup operation $m [ a ]_{\Gamma }$ that yields the subtree at address *a* in the memory *m*. The lookup function is partial, it will fail in case *a* is end-of-array or violates effective types. We first define the counterpart of lookup on memory trees and then lift it to memories.

#### **Definition 6.32**

The *lookup operation on memory trees*  is defined as:


The lookup operation uses the annotations $q \in \{\circ ,\bullet \}$ on  to give a formal semantics to the *strict-aliasing restrictions* [27, 6.5.2.3].

- The annotation $q = \bullet$ allows a union to be accessed via a reference whose variant is unequal to the current one. This is called type-punning.
- The annotation $q = \circ$ allows a union to be accessed only via a reference whose variant is equal to the current one. This means, it rules out type-punning.

Failure of type-punning is captured by partiality of the lookup operation. The behavior of type-punning of $\mathsf{union}_{t}\,(j,w,{\mathbf {{b}}})$ via a reference to variant *i* is described by the conversion $((\overline{w}\,{\mathbf {{b}}})_{[0,\,\mathsf{bitsizeof}_{\Gamma }\,\tau _i)})^{\tau _i}_{\Gamma }$. The memory tree *w* is converted into bits and reinterpreted as a memory tree of type $\tau _i$.

#### **Definition 6.33**

The *lookup operation on memories*  is defined as:provided that ${m}\,(\mathsf{index}\;a) = (w,\,\mu )$. In omitted cases the result is $\bot$. In this definition we let $i :=\mathsf{byte}_{\Gamma }\,a \cdot \mathsf{char\_bits}$ and $j :=(\mathsf{byte}_{\Gamma }\,a+1) \cdot \mathsf{char\_bits}$.

We have to take special care of addresses that refer to individual bytes rather than whole objects. Consider:
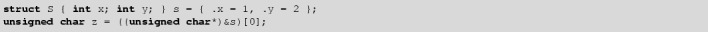


In this code, we obtain the first byte ((unsigned char*)&s)[0] of the struct s. This is formalized by flattening the entire memory tree of the struct s, and selecting the appropriate byte.

The C11 standard’s description of effective types [27, 6.5p6-7] states that an access (which is either a read or store) affects the effective type of the accessed object. This means that although reading from memory does not affect the memory contents, it may still affect the effective types. Let us consider an example where it is indeed the case that effective types are affected by a read:
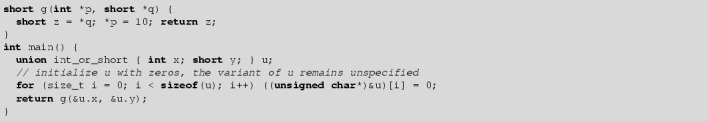


In this code, the variant of the union u is initially unspecified. The read *q in g *forces* its variant to y, making the assignment *p to variant x undefined. Note that it is important that we also assign undefined behavior to this example, a compiler may assume p and q to not alias regardless of how g is called.

We factor these side-effects out using a function $\mathsf{force}_{\Gamma }: \mathsf{addr}\rightarrow \mathsf{mem}\rightarrow \mathsf{mem}$ that updates the effective types (that is the variants of unions) after a successful lookup. The $\mathsf{force}_{\Gamma }$ function, as defined in Definition 6.5, can be described in terms of the alter operation $m[a / f]_{\Gamma }$ that applies the function $f : \mathsf{mtree}\rightarrow \mathsf{mtree}$ to the object at address *a* in the memory *m* and update variants of unions accordingly to *a*. To define $\mathsf{force}_{\Gamma }$ we let *f* be the identify.

#### **Definition 6.34**

Given a function $f : \mathsf{mtree}\rightarrow \mathsf{mtree}$, the *alter operation on memory trees*  is defined as:In the last two cases we have ${\Gamma }\,{t}= {\tau }$, $s :=\mathsf{bitsizeof}_{\Gamma }\,\tau _i$ and $z :=\mathsf{bitsizeof}_{\Gamma }\,(\mathsf{union}\;t)$. The result of $w[{{r}} / f]_{\Gamma }$ is only well-defined in case $w [ {{r}} ]_{\Gamma } \ne \bot$.

#### **Definition 6.35**

Given a function $f : \mathsf{mtree}\rightarrow \mathsf{mtree}$, the *alter operation on memories*  is defined as:provided that ${m}\,(\mathsf{index}\;a) = (w,\,\mu )$. In this definition we let:

$$\begin{aligned} \overline{f}\,w :=(\overline{w}_{[0,\,i)}\; {\overline{f\;{(\overline{w}_{[i,\,j)})^{\mathsf{unsigned}\;\mathsf{char}}_{\Gamma }}}}\; {\overline{w}_{[j,\,\mathsf{bitsizeof}_{\Gamma }\,(\mathsf{typeof}\;w))}})^{\mathsf{typeof}\;w}_{\Gamma } \end{aligned}$$

where $$i :=\mathsf{byte}_{\Gamma }\,a \cdot \mathsf{char\_bits}$$ and $$j :=(\mathsf{byte}_{\Gamma }\,a+1) \cdot \mathsf{char\_bits}$$.

The lookup and alter operation enjoy various properties; they preserve typing and satisfy laws about their interaction. We list some for illustration.

#### **Lemma 6.36**

(Alter commutes) If $\Gamma ,\Delta \vdash m$,  with:

- $$\Gamma ,\Delta \vdash a_1 : \tau _1$$, $$m [ a_1 ]_{\Gamma } = w_1$$, and $$\Gamma ,\Delta \vdash {f_1\,w_1} : {\tau _1}$$, and
- $$\Gamma ,\Delta \vdash a_2 : \tau _2$$, $$m [ a_2 ]_{\Gamma } = w_2$$, and $$\Gamma ,\Delta \vdash {f_2\,w_2} : {\tau _2}$$,

then we have:

$$\begin{aligned} m[a_2 / f_2]_{\Gamma }[a_1 / f_1]_{\Gamma } = m[a_1 / f_1]_{\Gamma }[a_2 / f_2]_{\Gamma }. \end{aligned}$$

#### **Lemma 6.37**

If $\Gamma ,\Delta \vdash m$, $m [ a ]_{\Gamma } = w$, and *a* is not a byte address, then:

$$\begin{aligned} (m[a / f]_{\Gamma }) [ a ]_{\Gamma } = f\,w. \end{aligned}$$

A variant of Lemma 6.37 for byte addresses is more subtle because a byte address can be used to modify padding. Since modifications of padding are masked, a successive lookup may yield a memory tree with more indeterminate bits. In Sect. 7.2 we present an alternative lemma that covers this situation.

We conclude this section with a useful helper function that *zips* a memory tree and a list. It is used in for example Definitions 6.58 and 7.22.

#### **Definition 6.38**

Given a function $f : \mathsf{pbit}\rightarrow B \rightarrow \mathsf{pbit}$, the operation that zips the leaves $\hat{f} : \mathsf{mtree}\rightarrow \mathsf{list}\;B \rightarrow \mathsf{mtree}$ is defined as:


### Representation of Values

Memory trees (Definition 6.23) are still rather low-level and expose permissions and implementation specific properties such as bit representations. In this section we define *abstract values*, which are like memory trees but have mathematical integers and pointers instead of bit representations as leaves. Abstract values are used in the external interface of the memory model.

#### **Definition 6.39**

*Base values* are inductively defined as:


While performing byte-wise operations (for example, byte-wise copying a struct containing pointer values), abstraction is broken, and pointer fragment bits have to reside outside of memory. The value $\mathsf{byte}\,{{{b}}}$ is used for this purpose.

#### **Definition 6.40**

The judgment $\Gamma ,\Delta \vdash _{\mathsf b} {v}_{\mathsf{b}} : {\tau }_{\mathsf{b}}$ describes that *the base value* ${v}_{\mathsf{b}}$ *has base type* ${\tau }_{\mathsf{b}}$. It is inductively defined as:


The side-conditions of the typing rule for $\mathsf{byte}\,{{{b}}}$ ensure canonicity of representations of base values. It ensures that the construct $\mathsf{byte}\,{{{b}}}$ is only used if ${{b}}$ cannot be represented as an integer $\mathsf{int}_{\mathsf{unsigned}\;\mathsf{char}}\,{x}$ or $\mathsf{indet}\,({\mathsf{unsigned}\;\mathsf{char}})$.

In Definition 6.44 we define abstract values by extending base values with constructs for arrays, structs and unions. In order to define the operations to look up and store values in memory, we define conversion operations between abstract values and memory trees. Recall that the leaves of memory trees, which represent base values, are just sequences of bits. We therefore first define operations that convert base values to and from bits. These operations are called flatten and unflatten.

#### **Definition 6.41**

The *flatten operation*  is defined as:The operation  is defined in Definition 4.4.

#### **Definition 6.42**

The *unflatten operation* $(\_)^{\Gamma }_{{\tau }_{\mathsf{b}}}: \mathsf{list}\;\mathsf{bit}\rightarrow \mathsf{baseval}$ is defined as:The operation $(\_)_{{\tau }_{\mathsf{i}}} : \mathsf{list}\;\mathsf{bool}\rightarrow \mathbb {Z}$ is defined in Definition 4.4.

The encoding of pointers is an important aspect of the flatten operation related to our treatment of effective types. Pointers are encoded as sequences of *frozen* pointer fragment bits $(\mathsf{ptr}\;{|\,p\,|_\circ })_{{i}}$ (see Definition 6.5 for the definition of frozen pointers). Recall that the flatten operation is used to store base values in memory, whereas the unflatten operation is used to retrieve them. This means that whenever a pointer *p* is stored and read back, the frozen variant $|\,p\,|_\circ$ is obtained.

#### **Lemma 6.43**

For each $\Gamma ,\Delta \vdash _{\mathsf b} {v}_{\mathsf{b}} : {\tau }_{\mathsf{b}}$ we have .

Freezing formally describes the situations in which type-punning is allowed since a frozen pointer cannot be used to access a union of another variant than its current one (Definition 6.32). Let us consider an example:
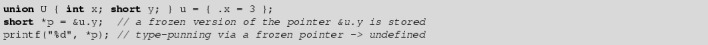


Here, an attempt to type-punning is performed via the frozen pointer p, which is formally represented as:The lookup operation on memory trees (which will be used to obtain the value of *p from memory, see Definitions 6.32 and 6.58) will fail. The annotation $\circ$ prevents a union from being accessed through an address to another variant than its current one. In the example below type-punning is allowed:
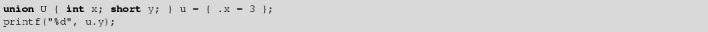


Here, type-punning is allowed because it is performed directly via u.y, which has not been stored in memory, and thus has not been frozen.

#### **Definition 6.44**

*Abstract values* are inductively defined as:


The abstract value $\overline{\mathsf{union}}_{t}\,{{{v}}}$ represents a union whose variant is unspecified. The values ${{v}}$ correspond to interpretations of *all* variants of $\mathsf{union}\;t$. Consider:
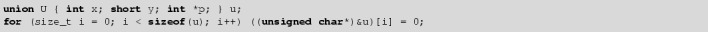


Here, the object representation of u is initialized with zeros, and its variant thus remains unspecified. The abstract value of u is^5^:

$$\begin{aligned} \overline{\mathsf{union}}_{\mathtt U}\,{[\, {\mathsf{int}_{{{{\mathsf{signed}\;\mathsf{int}}}}}\,{0}},\ {\mathsf{int}_{{\mathsf{signed}\;\mathsf{short}}}\,{0}},\ {\mathsf{indet}\,({{{{{\mathsf{signed}\;\mathsf{int}}}}}}{*})} \,]} \end{aligned}$$

Recall that the variants of a union occupy a single memory area, so the sequence ${{v}}$ of a union value $\overline{\mathsf{union}}_{t}\,{{{v}}}$ cannot be arbitrary. There should be a common bit sequence representing it. This is not the case in:

$$\begin{aligned} \overline{\mathsf{union}}_{\mathtt U}\,{[\, {\mathsf{int}_{{{{\mathsf{signed}\;\mathsf{int}}}}}\,{0}},\ {\mathsf{int}_{{\mathsf{signed}\;\mathsf{short}}}\,{1}},\ {\mathsf{indet}\,({{{{{\mathsf{signed}\;\mathsf{int}}}}}}{*})} \,]} \end{aligned}$$

The typing judgment for abstract values guarantees that ${{v}}$ can be represented by a common bit sequence. In order to express this property, we first define the unflatten operation that converts a bit sequence into an abstract value.

#### **Definition 6.45**

The *unflatten operation* $(\_)^{\tau }_{\Gamma }: \mathsf{list}\;\mathsf{bit}\rightarrow \mathsf{val}$ is defined as:


#### **Definition 6.46**

The judgment $\Gamma ,\Delta \vdash v : \tau$ describes that *the value* *v* has type $\tau$. It is inductively defined as:


The flatten operation , which converts an abstract value *v* into a bit representation , is more difficult to define (we need this operation to define the conversion operation from abstract values into memory trees, see Definition 6.49). Since padding bits are not present in abstract values, we have to insert these. Also, in order to obtain the bit representation of an unspecified $\overline{\mathsf{union}}_{t}\,{{{v}}}$ value, we have to *construct* the common bit sequence ${{b}}$ representing ${{v}}$. The typing judgment guarantees that such a sequence exists, but since it is not explicit in the value $\overline{\mathsf{union}}_{t}\,{{{v}}}$, we have to reconstruct it from ${{v}}$. Consider:
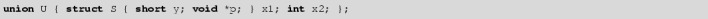


Assuming $\mathsf{sizeof}_{\Gamma }\;({{\mathsf{signed}\;\mathsf{int}}}) = \mathsf{sizeof}_{\Gamma }\;({\mathsf{any}{*}}) = 4$ and $\mathsf{sizeof}_{\Gamma }\;({{\mathsf{signed}\;\mathsf{short}}}) = 2$, a well-typed $\mathsf{union}\;\mathtt U$ value of an unspecified variant may be:

$$\begin{aligned} v = \overline{\mathsf{union}}_{\mathtt U}\,{[\, \mathsf{struct}_{\mathtt S}\,{[\, {\mathsf{int}_{\mathsf{signed}\;\mathsf{short}}\,{0}}, {\mathsf{ptr}\,{p}} \,]}, {\mathsf{int}_{\mathsf{signed}\;\mathsf{int}}\,{0}} \,]}. \end{aligned}$$

The flattened versions of the variants of *v* are:This example already illustrates that so as to obtain the common bit sequence  of *v* we have to insert padding bits and “join” the padded bit representations.

#### **Definition 6.47**

The *join operation on bits* $\sqcup : \mathsf{bit}\rightarrow \mathsf{bit}\rightarrow \mathsf{bit}$ is defined as:


#### **Definition 6.48**

The *flatten operation*  is defined as:


The operation $\mathsf{ofval}_{\Gamma }: \mathsf{list}\;\mathsf{perm}\rightarrow \mathsf{val}\rightarrow \mathsf{mtree}$, which converts a value *v* of type $\tau$ into a memory tree $\mathsf{ofval}_{\Gamma }\,{\gamma }\,v$, is albeit technical fairly straightforward. In principle it is just a recursive definition that uses the flatten operation  for base values ${{v}_{\mathsf{b}}}$ and the flatten operation  for unions $\overline{\mathsf{union}}_{t}\,{{{v}}}$ of an unspecified variant.

The technicality is that abstract values do not contain permissions, so we have to merge the given value with permissions. The sequence ${\gamma }$ with $|{\gamma }| = \mathsf{bitsizeof}_{\Gamma }\,\tau$ represents a flattened sequence of permissions. In the definition of the memory store $m \langle a := v \rangle _{\Gamma }$ (see Definition 6.58), we convert *v* into the stored memory tree $\mathsf{ofval}_{\Gamma }\,{\gamma }\,v$ where $\gamma$ constitutes the old permissions of the object at address *a*.

#### **Definition 6.49**

The operation $\mathsf{ofval}_{\Gamma }: \mathsf{list}\;\mathsf{perm}\rightarrow \mathsf{val}\rightarrow \mathsf{mtree}$ is defined as:


Converting a memory tree into a value is as expected: permissions are removed and unions are interpreted as values corresponding to each variant.

#### **Definition 6.50**

The operation $\mathsf{toval}_{\Gamma }: \mathsf{mtree}\rightarrow \mathsf{val}$ is defined as:


The function $\mathsf{toval}_{\Gamma }$ is an inverse of $\mathsf{ofval}_{\Gamma }$ up to freezing of pointers. Freezing is intended, it makes indirect type-punning illegal.

#### **Lemma 6.51**

Given $\Gamma ,\Delta \vdash v : \tau$, and let ${\gamma }$ be a flattened sequence of permissions with $|{\gamma }| = \mathsf{bitsizeof}_{\Gamma }\,\tau$, then we have:

$$\begin{aligned} \mathsf{toval}_{\Gamma }\,(\mathsf{ofval}_{\Gamma }\,{\gamma }\,v) = |\,v\,|_\circ . \end{aligned}$$

The other direction does not hold because invalid bit representations will become indeterminate values.
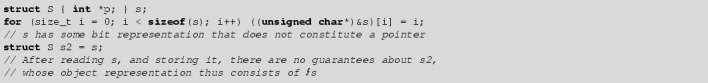


We finish this section by defining the indeterminate abstract value $\mathsf{new}_{\Gamma }\,\tau$, which consists of indeterminate base values. The definition is similar to its counterpart on memory trees (Definition 6.31).

#### **Definition 6.52**

The operation $\mathsf{new}_{\Gamma }\,: \mathsf{type}\rightarrow \mathsf{val}$ that yields the indeterminate value is defined as:


#### **Lemma 6.53**

If $$\Gamma \vdash {\tau }$$, then:

$$\begin{aligned} \mathsf{toval}_{\Gamma }\,(\mathsf{new}_{\Gamma }^{\gamma }\,\tau ) = \mathsf{new}_{\Gamma }\,\tau \quad \mathrm{and}\quad \mathsf{ofval}_{\Gamma }\,(\gamma ^ {\mathsf{bitsizeof}_{\Gamma }\,\tau })\,(\mathsf{new}_{\Gamma }\,\tau ) = \mathsf{new}_{\Gamma }^{\gamma }\,\tau . \end{aligned}$$

### Memory Operations

Now that we have all primitive definitions in place, we can compose these to implement the actual memory operations as described in the beginning of this section. The last part that is missing is a data structure to keep track of objects that have been locked. Intuitively, this data structure should represent a set of addresses, but up to overlapping addresses.

#### **Definition 6.54**

*Locksets* are defined as:


Elements of locksets are pairs $(o,\,i)$ where $o \in \mathsf{index}$ describes the object identifier and $i \in \mathbb {N}$ a bit-offset in the object described by *o*. We introduce a typing judgment to describe that the structure of locksets matches up with the memory layout.

#### **Definition 6.55**

The judgment $\Gamma ,\Delta \vdash \Omega$ describes that *the lockset* $\Omega$ *is valid*. It is inductively defined as:


#### **Definition 6.56**

The *singleton lockset* $\{ \_ \}_{\Gamma }: \mathsf{addr}\rightarrow \mathsf{lockset}$ is defined as:

$$\begin{aligned} \{ a \}_{\Gamma } :=\{ (\mathsf{index}\;a, i) \;|\;\mathsf{bitoffset}_{\Gamma }\,a \le i < \mathsf{bitoffset}_{\Gamma }\,a + \mathsf{bitsizeof}_{\Gamma }\,(\mathsf{typeof}\;a) \}. \end{aligned}$$

#### **Lemma 6.57**

If $\Gamma ,\Delta \vdash a_1 : \sigma _1$ and $\Gamma ,\Delta \vdash a_2 : \sigma _2$ and $\Gamma \vdash \{a_1,a_2\}\ \mathsf{strict}$, then:


#### **Definition 6.58**

The *memory operations* are defined as:


The lookup operation $m \langle a \rangle _{\Gamma }$ uses the lookup operation $m [ a ]_{\Gamma }$ that yields a memory tree *w* (Definition 6.33), and then converts *w* into the value $\mathsf{toval}_{\Gamma }\,w$. The operation $m [ a ]_{\Gamma }$ already yields $\bot$ in case effective types are violated or *a* is an end-of-array address. The additional condition of $m \langle a \rangle _{\Gamma }$ ensures that the permissions allow for a read access. Performing a lookup affects the effective types of the object at address *a*. This is factored out by the operation $\mathsf{force}_{\Gamma }\,a\;m$ which applies the identity function to the subobject at address *a* in the memory *m*. Importantly, this does not change the memory contents, but merely changes the variants of the involved unions.

The store operation $m \langle a := v \rangle _{\Gamma }$ uses the alter operation $m[a / \lambda w \,.\,\mathsf{ofval}_{\Gamma }\,({\overline{w}}_{\mathbf {1}})\,v]_{\Gamma }$ on memories (Definition 6.35) to apply $\lambda w \,.\,\mathsf{ofval}_{\Gamma }\,({\overline{w}}_{\mathbf {1}})\,v$ to the subobject at address *a*. The stored value *v* is converted into a memory tree while retaining the permissions ${\overline{w}}_{\mathbf {1}}$ of the previously stored memory tree *w* at address *a*.

The definition of $\mathsf{lock}_{\Gamma }\,a\;m$ is straightforward. In the Coq development we use a map operation on memory trees to apply the function $\mathsf{lock}$ (Definition 5.5) to the permission of each bit of the memory tree at address *a*.

The operation $\mathsf{unlock}\;\Omega \;m$ unlocks a whole lockset $\Omega$, rather than an individual address, in memory *m*. For each memory tree *w* at object identifier *o*, it converts $\Omega$ to a Boolean vector ${{y}} = ((o,\,0) \in \Omega ) \ldots ((o,\,|\mathsf{bitsizeof}_{\Gamma }\,(\mathsf{typeof}\;w)| - 1) \in \Omega )$ and merges *w* with ${{y}}$ (using Definition 6.38) to apply $\mathsf{unlock}$ (Definition 5.5) to the permissions of bits that should be unlocked in *w*. We show some lemmas to illustrate that the operations for locking and unlocking enjoy the intended behavior:

#### **Lemma 6.59**

If $\Gamma ,\Delta \vdash m$ and $\Gamma ,\Delta \vdash a : {\tau }$ and $\mathsf{writable}_{\Gamma }\,a\;m$, then we have:

$$\begin{aligned} \mathsf{locks}\;(\mathsf{lock}_{\Gamma }\,a\;m) = \mathsf{locks}\;m \cup \{ a \}_{\Gamma }. \end{aligned}$$

#### **Lemma 6.60**

If $$\Omega \subseteq \mathsf{locks}\;m$$, then $$\mathsf{locks}\;(\mathsf{unlock}\;\Omega \;m) = \mathsf{locks}\;m \setminus \Omega $$.

Provided $o \notin \mathsf{dom}\;m$, allocation $\mathsf{alloc}_{\Gamma }\,o\;v\;\mu \;m$ extends the memory with a new object holding the value *v* and *full* permissions . Typically we use $v = \mathsf{new}_{\Gamma }\,\tau$ for some $\tau$, but global and static variables are allocated with a specific value *v*.

The operation $\mathsf{free}\;o\;m$ deallocates the object *o* in *m*, and keeps track of the type of the deallocated object. In order to deallocate dynamically obtained memory via free, the side-condition $\mathsf{freeable}\;a\;m$ describes that the permissions are sufficient for deallocation, and that *a* points to the first element of a malloced array.

All operations preserve typing and satisfy the expected laws about their interaction. We list some for illustration.

#### **Fact 6.61**

If $\mathsf{writable}_{\Gamma }\,a\;m$, then there exists a value *v* with $a \langle m \rangle _{\Gamma } = v$.

#### **Lemma 6.62**

(Stores commute) If $\Gamma ,\Delta \vdash m$ and  with:

- $$\Gamma ,\Delta \vdash a_1 : \tau _1$$, $$\mathsf{writable}_{\Gamma }\,a_1\;m$$, and $$\Gamma ,\Delta \vdash {v_1} : {\tau _1}$$, and
- $$\Gamma ,\Delta \vdash a_2 : \tau _2$$, $$\mathsf{writable}_{\Gamma }\,a_2\;m$$, and $$\Gamma ,\Delta \vdash {v_2} : {\tau _2}$$,

then we have:

$$\begin{aligned} m \langle a_2 := v_2 \rangle _{\Gamma } \langle a_1 := v_1 \rangle _{\Gamma } = m \langle a_1 := v_1 \rangle _{\Gamma } \langle a_2 := v_2 \rangle _{\Gamma }. \end{aligned}$$

#### **Lemma 6.63**

(Lookup after store) If $\Gamma ,\Delta \vdash m$ and $\Gamma ,\Delta \vdash a : \tau$ and $\Gamma ,\Delta \vdash v : \tau$ and $\mathsf{writable}_{\Gamma }\,a\;m$ and *a* is not a byte address, then we have:

$$\begin{aligned} (m \langle a := v \rangle _{\Gamma }) \langle a \rangle _{\Gamma } = |\,v\,|_\circ . \end{aligned}$$

Storing a value *v* in memory and then retrieving it, does not necessarily yield the same value *v*. It intentionally yields the value $|\,v\,|_\circ$ whose pointers have been frozen. Note that the above result does not hold for byte addresses, which may store a value in a padding byte, in which case the resulting value is indeterminate.

#### **Lemma 6.64**

(Stores and lookups commute) If $\Gamma ,\Delta \vdash m$ and  and $\Gamma ,\Delta \vdash a_2 : \tau _2$ and $\mathsf{writable}_{\Gamma }\,a_2\;m$ and $\Gamma ,\Delta \vdash v_2 : \tau _2$, then we have:

$$\begin{aligned} m \langle a_1 \rangle _{\Gamma } = v_1 \quad \hbox {implies}\quad (m \langle a_2 := v_2 \rangle _{\Gamma }) \langle a_1 \rangle _{\Gamma } = v_1. \end{aligned}$$

These results follow from Lemmas 6.36, 6.37 and 6.51.

## Formal Proofs

### Type-Based Alias Analysis

The purpose of C11’s notion of effective types [27, 6.5p6-7] is to make it possible for compilers to perform typed-based alias analysis. Consider:
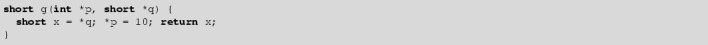


Here, a compiler should be able to assume that p and q are not aliased because they point to objects with different types (although the integer types $\mathsf{signed}\;\mathsf{short}$ and $\mathsf{signed}\;\mathsf{int}$ may have the same representation, they have different integer ranks, see Definition 4.2, and are thus different types). If g is called with aliased pointers, execution of the function body should have undefined behavior in order to allow a compiler to soundly assume that p and q are not aliased.

From the C11 standard’s description of effective types it is not immediate that calling g with aliased pointers results in undefined behavior. We prove an abstract property of our memory model that shows that this is indeed a consequence, and that indicates a compiler can perform type-based alias analysis. This also shows that our interpretation of effective types of the C11 standard, in line with the interpretation from the GCC documentation [20], is sensible.

#### **Definition 7.1**

A type $\tau$ is a *subobject type of* $\sigma$, notation $\tau \subseteq _{\Gamma } \sigma$, if there exists some reference $r$ with $\Gamma \vdash {{r}} : \sigma \rightarrowtail \tau$.

For example, int[2] is a subobject type of struct S { int x[2]; int y[3]; } and int[2][2], but not of struct S { short x[2]; }, nor of int(*)[2].

#### **Theorem 7.2**

(Strict-aliasing) Given $\Gamma ,\Delta \vdash m$, frozen addresses $a_1$ and $a_2$ with $\Delta ,m \vdash a_1 : \sigma _1$ and $\Delta ,m \vdash a_2 : \sigma _2$ and $\sigma _1, \sigma _2 \ne {{\mathsf{unsigned}\;\mathsf{char}}}$, then either:

1. We have $\sigma _1 \subseteq _{\Gamma } \sigma _2$ or $\sigma _2 \subseteq _{\Gamma } \sigma _1$.
2. We have .
3. Accessing $a_1$ after accessing $a_2$ and *vice versa* fails. That means:
  - $$(\mathsf{force}_{\Gamma }\,a_2\;m) \langle a_1 \rangle _{\Gamma } = \bot $$ and $$(\mathsf{force}_{\Gamma }\,a_1\;m) \langle a_2 \rangle _{\Gamma } = \bot $$, and
  - $m \langle a_2 := v_1 \rangle _{\Gamma } \langle a_1 \rangle _{\Gamma } = \bot$ and $m \langle a_1 := v_2 \rangle _{\Gamma } \langle a_2 \rangle _{\Gamma } = \bot$ for all stored values $v_1$ and $v_2$.

This theorem implies that accesses to addresses of disjoint type are either non-overlapping or have undefined behavior. Fact 6.61 accounts for a store after a lookup. Using this theorem, a compiler can optimize the generated code in the example based on the assumption that p and q are not aliased. Reconsider:
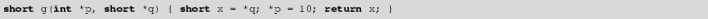


If p and q are aliased, then calling g yields undefined behavior because the assignment *p = 10 violates effective types. Let *m* be the initial memory while executing g, and let $a_{\mathtt p}$ and $a_{\mathtt q}$ be the addresses corresponding to p and q, then the condition $\mathsf{writable}_{\Gamma }\,a_{\mathtt p}\;(\mathsf{force}_{\Gamma }\,a_{\mathtt q}\;m)$ does not hold by Theorem 7.2 and Fact 6.61.

### Memory Refinements

This section defines the notion of *memory refinements* that allows us to relate memory states. The author’s PhD thesis [33] shows that the $\mathrm{CH}_2\mathrm{O}$ operational semantics is invariant under this notion. Memory refinements form a general way to validate many common-sense properties of the memory model in a formal way. For example, they show that the memory is invariant under relabeling. More interestingly, they show that symbolic information (such as variants of unions) cannot be observed.

Memory refinements also open the door to reason about program transformations. We demonstrate their usage by proving soundness of constant propagation and by verifying an abstract version of memcpy.

Memory refinements are a variant of Leroy and Blazy’s notion of memory extensions and injections [41]. A memory refinement is a relation $m_1 \sqsubseteq _{\Gamma }^{f} m_2$ between a source memory state $m_1$ and target memory state $m_2$, where:

1. The function $f : \mathsf{index}\rightarrow \mathsf{option}\;(\mathsf{index}\times \mathsf{ref})$ is used to rename object identifiers and to coalesce multiple objects into subobjects of a compound object.
2. Deallocated objects in $m_1$ may be replaced by arbitrary objects in $m_2$.
3. Indeterminate bits  in $m_1$ may be replaced by arbitrary bits in $m_2$.
4. Pointer fragment bits ${(\mathsf{ptr}\;{p})_{{i}}}$ that belong to deallocated pointers in $m_1$ may be replaced by arbitrary bits in $m_2$.
5. Effective types may be weakened. That means, unions with a specific variant in $m_1$ may be replaced by unions with an unspecified variant in $m_2$, and pointers with frozen union annotations $\circ$ in $m_1$ may be replaced by pointers with unfrozen union annotations $\bullet$ in $m_2$.

The key property of a memory refinement $m_1 \sqsubseteq _{\Gamma }^{f} m_2$, as well as of Leroy and Blazy’s memory extensions and injections, is that memory operations are more defined on the target memory $m_2$ than on the source memory $m_1$. For example, if a lookup succeeds on $m_1$, it also succeed on $m_2$ and yield a related value.

The main judgment $m_1 \sqsubseteq _{\Gamma }^{f : \Delta _1 \mapsto \Delta _2} m_2$ of memory refinements will be built using a series of refinement relations on the structures out of which the memory consists (addresses, pointers, bits, memory trees, values). All of these judgments should satisfy some basic properties, which are captured by the judgment $\Delta _1 \sqsubseteq _{\Delta }^{f} \Delta _2$.

#### **Definition 7.3**

A *renaming function* $f : \mathsf{index}\rightarrow \mathsf{option}\;(\mathsf{index}\times \mathsf{ref})$ is a *refinement*, notation $\Delta _1 \sqsubseteq _{\Delta }^{f} \Delta _2$, if the following conditions hold:

1. If $f\,o_1 = (o,\,{{r}}_1)$ and $f\,o_2 = (o,\,{{r}}_2)$, then $o_1 = o_2$ or  (*injectivity*).
2. If $$f\,o_1 = (o_2,\,{{r}})$$, then $$\mathsf{frozen}\;{{r}}$$.
3. If $f\,o_1 = (o_2,\,{{r}})$ and $\Delta _1 \vdash o_1 : \sigma$, then $\Delta _2 \vdash o_2 : \tau$ and $\Gamma \vdash {{r}} : \tau \rightarrowtail \sigma$ for a $\tau$.
4. If $f\,o_1 = (o_2,\,{{r}})$ and $\Delta _2 \vdash o_2 : \tau$, then $\Delta _1 \vdash o_1 : \sigma$ and $\Gamma \vdash {{r}} : \tau \rightarrowtail \sigma$ for a $\sigma$.
5. If $f\,o_1 = (o_2,\,{{r}})$ and $\Delta _1 \vdash o_1\ \mathsf{alive}$, then $\Delta _2 \vdash o_2\ \mathsf{alive}$.

The renaming function $f : \mathsf{index}\rightarrow \mathsf{option}\;(\mathsf{index}\times \mathsf{ref})$ is the core of all refinement judgments. It is used to rename object identifiers and to coalesce multiple source objects into subobjects of a single compound target object.

Consider a renaming *f* with  and , and an environment $\Gamma$ with ${\Gamma }\,{t}= [\, \tau _1,\tau _2 \,]$. This gives rise to following refinement:


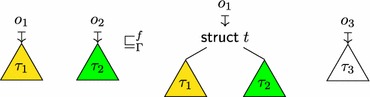


Injectivity of renaming functions guarantees that distinct source objects are coalesced into disjoint target subobjects. In the case of Blazy and Leroy, the renaming functions have type $\mathsf{index}\rightarrow \mathsf{option}\;(\mathsf{index}\times \mathbb {N})$, but we replaced the natural number by a reference since our memory model is structured using trees.

Since memory refinements rearrange the memory layout, addresses should be rearranged accordingly. The judgment $a_1 \sqsubseteq _{\Gamma }^{f : \Delta _1 \mapsto \Delta _2} a_2 : {\tau }_{\mathsf{p}}$ describes how $a_2$ is obtained by renaming $a_1$ according to the renaming *f*, and moreover allows frozen union annotations $\circ$ in $a_1$ to be changed into unfrozen ones $\bullet$ in $a_2$. The index ${\tau }_{\mathsf{p}}$ in the judgment $a_1 \sqsubseteq _{\Gamma }^{f : \Delta _1 \mapsto \Delta _2} a_2 : {\tau }_{\mathsf{p}}$ corresponds to the type of $a_1$ and $a_2$.

The judgment for addresses is lifted to the judgment for pointers in the obvious way. The judgment for bits is inductively defined as:The last two rules allow indeterminate bits , as well as pointer fragment bits ${(\mathsf{ptr}\;{{a}})_{{i}}}$ belonging to deallocated storage, to be replaced by arbitrary bits *b*.

The judgment is lifted to memory trees following the tree structure and using the following additional rule:This rule allows a union that has a specific variant in the source to be replaced by a union with an unspecified variant in the target. The direction seems counter intuitive, but keep in mind that unions with an unspecified variant allow more behaviors.

#### **Lemma 7.4**

If $w_1 \sqsubseteq _{\Gamma }^{f : \Delta _1 \mapsto \Delta _2} w_2 : \tau$, then $\Gamma ,\Delta _1 \vdash {w_1} : {\tau }$ and $\Gamma ,\Delta _2 \vdash {w_2} : {\tau }$.

This lemma is useful because it removes the need for simultaneous inductions on both typing and refinement judgments.

We define $m_1 \sqsubseteq _{\Gamma }^{f} m_2$ as $m_1 \sqsubseteq _{\Gamma }^{f : \overline{m_1} \mapsto \overline{m_2}} m_2$, where the judgment $m_1 \sqsubseteq _{\Gamma }^{f : \Delta _1 \mapsto \Delta _2} m_2$ is defined such that if $f\,o_1 = (o_2,\,{{r}})$, then:


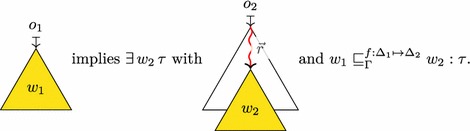


The above definition makes sure that objects are renamed, and possibly coalesced into subobjects of a compound object, as described by the renaming function *f*.

In order to reason about program transformations modularly, we show that memory refinements can be composed.

#### **Lemma 7.5**

Memory refinements are reflexive for valid memories, that means, if $\Gamma ,\Delta \vdash m$, then $m \sqsubseteq _{\Gamma }^{\mathsf{id} : \Delta \mapsto \Delta } m$ where $\mathsf{id}\,o :=(o,\,\varepsilon )$.

#### **Lemma 7.6**

Memory refinements compose, that means, if $m_1 \sqsubseteq _{\Gamma }^{f : \Delta _1 \mapsto \Delta _2} m_2$ and $m_2 \sqsubseteq _{\Gamma }^{f' : \Delta _2 \mapsto \Delta _3} m_3$, then  where:


All memory operations are preserved by memory refinements. This property is not only useful for reasoning about program transformations, but also indicates that the memory interface does not expose internal details (such as variants of unions) that are unavailable in the memory of a (concrete) machine.

#### **Lemma 7.7**

If $m_1 \sqsubseteq _{\Gamma }^{f : \Delta _1 \mapsto \Delta _2} m_2$ and $a_1 \sqsubseteq _{\Gamma }^{f : \Delta _1 \mapsto \Delta _2} a_2 : \tau$ and $m_1 \langle a_1 \rangle _{\Gamma } = v_1$, then there exists a value $v_2$ with $m_2 \langle a_2 \rangle _{\Gamma } = v_2$ and $v_1 \sqsubseteq _{\Gamma }^{f : \Delta _1 \mapsto \Delta _2} v_2 : \tau$.

#### **Lemma 7.8**

If $m_1 \sqsubseteq _{\Gamma }^{f : \Delta _1 \mapsto \Delta _2} m_2$ and $a_1 \sqsubseteq _{\Gamma }^{f : \Delta _1 \mapsto \Delta _2} a_2 : \tau$ and $v_1 \sqsubseteq _{\Gamma }^{f : \Delta _1 \mapsto \Delta _2} v_2 : \tau$ and $\mathsf{writable}_{\Gamma }\,m_1\;a_1$, then:

1. We have $$\mathsf{writable}_{\Gamma }\,m_2\;a_2$$.
2. We have $$m_1 \langle a_1 := v_1 \rangle _{\Gamma } \sqsubseteq _{\Gamma }^{f : \Delta _1 \mapsto \Delta _2} m_2 \langle a_2 := v_2 \rangle _{\Gamma }$$.

As shown in Lemma 6.63, storing a value *v* in memory and then retrieving it, does not necessarily yield the same value *v*. In case of a byte address, the value may have been stored in padding and therefore have become indeterminate. Secondly, it intentionally yields the value $|\,v\,|_\circ$ in which all pointers are frozen. However, the widely used compiler optimization of constant propagation, which substitutes values of known constants at compile time, is still valid in our memory model.

#### **Lemma 7.9**

If $$\Gamma ,\Delta \vdash v : \tau $$, then $$|\,v\,|_\circ \sqsubseteq _{\Gamma }^{\Delta } v : \tau $$.

#### **Theorem 7.10**

(Constant propagation) If $\Gamma ,\Delta \vdash m$ and $\Gamma ,\Delta \vdash a : \tau$ and $\Gamma ,\Delta \vdash v : \tau$ and $\mathsf{writable}_{\Gamma }\,a\;m$, then there exists a value $v'$ with:

$$\begin{aligned} m \langle a := v \rangle _{\Gamma } \langle a \rangle _{\Gamma } = v' \quad \hbox {and}\quad v' \sqsubseteq _{\Gamma }^{\Delta } v : \tau . \end{aligned}$$

Copying an object *w* by an assignment results in it being converted to a value $\mathsf{toval}_{\Gamma }\,w$ and back. This conversion makes invalid representations of base values indeterminate. Copying an object *w* byte-wise results in it being converted to bits $\overline{w}$ and back. This conversion makes all variants of unions unspecified. The following theorem shows that a copy by assignment can be transformed into a byte-wise copy.

#### **Theorem 7.11**

(Memcpy) If $\Gamma ,\Delta \vdash {w} : {\tau }$, then:

$$\begin{aligned} \mathsf{ofval}_{\Gamma }\,({\overline{w}}_{\mathbf {1}})\,(\mathsf{toval}_{\Gamma }\,w) \sqsubseteq _{\Gamma }^{\Delta } w \sqsubseteq _{\Gamma }^{\Delta }(\overline{w})^{\tau }_{\Gamma } : \tau . \end{aligned}$$

Unused reads cannot be removed unconditionally in the $\mathrm{CH}_2\mathrm{O}$ memory model because these have side-effects in the form of uses of the $\mathsf{force}_{\Gamma }$ operation that updates effective types. We show that uses of $\mathsf{force}_{\Gamma }$ can be removed for frozen addresses.

#### **Theorem 7.12**

If $\Gamma ,\Delta \vdash m$ and $m \langle a \rangle _{\Gamma } \ne \bot$ and $\mathsf{frozen}\;a$, then $\mathsf{force}_{\Gamma }\,a\;m \sqsubseteq _{\Gamma }^{\Delta } m$.

### Reasoning About Disjointness

In order to prove soundness of the $\mathrm{CH}_2\mathrm{O}$ axiomatic semantics, we often needed to reason about preservation of disjointness under memory operations [33]. This section describes some machinery to ease reasoning about disjointness. We show that our machinery, as originally developed in [31], extends to any separation algebra.

#### **Definition 7.13**

*Disjointness of a list* ${{x}}$, notation $\bot \,{{x}}$, is defined as:

1. $$\bot \,\varepsilon $$
2. If $\bot \,{{x}}$ and $x\, {\mathrel {\bot }}\, \bigcup {{x}}$, then $\bot \,(x\,{{x}})$

Notice that $\bot \,{{x}}$ is stronger than having $x_i\, {\mathrel {\bot }}\, x_j$ for each $i \ne j$. For example, using fractional permissions, we do not have $\bot \,[\, 0.5,\,0.5,\,0.5 \,]$ whereas $0.5\, {\mathrel {\bot }}\, 0.5$ clearly holds. Using disjointness of lists we can for example state the associativity law (law 3 of Definition 5.1) in a symmetric way:

#### **Fact 7.14**

If $$\bot \,(x\;y\;z)$$, then $$x \,{\mathrel {\cup }}\, (y \,{\mathrel {\cup }}\, z) = (x \,{\mathrel {\cup }}\, y) \,{\mathrel {\cup }}\, z$$.

We define a relation  that expresses that ${{x}}_1$ and ${{x}}_2$ behave equivalently with respect to disjointness.

#### **Definition 7.15**

*Equivalence of lists* ${{x}}_1$ *and* ${{x}}_2$ *with respect to disjointness*, notation , is defined as:


It is straightforward to show that  is reflexive and transitive, is respected by concatenation of lists, and is preserved by list containment. Hence,  is an equivalence relation, a congruence with respect to concatenation of lists, and is preserved by permutations. The following results (on arbitrary separation algebras) allow us to reason algebraically about disjointness.

#### **Fact 7.16**

If , then $\bot \,{{x}}_1$ implies $\bot \,{{x}}_2$.

#### **Fact 7.17**

If , then $\bot \,{{x}}_1$ iff $\bot \,{{x}}_2$.

#### **Theorem 7.18**

We have the following algebraic properties:


In Sect. 7.4 we show that we have similar properties as the above for the specific operations of our memory model.

### The Memory as a Separation Algebra

We show that the $\mathrm{CH}_2\mathrm{O}$ memory model is a separation algebra, and that the separation algebra operations interact appropriately with the memory operations that we have defined in Sect. 6.

In order to define the separation algebra relations and operations on memories, we first define these on memory trees. Memory trees do not form a separation algebra themselves due to the absence of a unique $\emptyset$ element (memory trees have a distinct identity element $\mathsf{new}_{\Gamma }^{\tau }\,$ for each type $\tau$, see Definition 6.31). The separation algebra of memories is then defined by lifting the definitions on memory trees to memories (which are basically finite functions to memory trees).

#### **Definition 7.19**

The predicate $\mathsf{valid}: \mathsf{mtree}\rightarrow \mathsf{Prop}$ is inductively defined as:


#### **Fact 7.20**

If $$\Gamma ,\Delta \vdash {w} : {\tau }$$, then $$\mathsf{valid}\;w$$.

The $\mathsf{valid}$ predicate specifies the subset of memory trees on which the separation algebra structure is defined. The definition basically lifts the $\mathsf{valid}$ predicate from the leaves to the trees. The side-condition $\lnot \mathsf{unmapped}\;(\overline{w}\,{{\mathbf {{b}}}})$ on $\mathsf{union}_{t}\,(i,w,{\mathbf {{b}}})$ memory trees ensures canonicity, unions whose permissions are unmapped cannot be accessed and are thus kept in unspecified variant. Unmapped unions $\overline{\mathsf{union}}_{t}\,{{\mathbf {{b}}}}$ can be combined with other unions using . The rationale for doing so will become clear in the context of the separation logic in the author’s PhD thesis [33].

#### **Definition 7.21**

The relation  is inductively defined as:


#### **Definition 7.22**

The operation  is defined as:In the last two clauses,  is a modified version of the memory tree *w* in which the elements on the leaves of *w* are zipped with ${\mathbf {{b}}}$ using the  operation on permission annotated bits (see Definitions 6.38 and 5.13).

The definitions of $\mathsf{valid}$,  and  on memory trees satisfy all laws of a separation algebra (see Definition 5.1) apart from those involving $\emptyset$. We prove the cancellation law explicitly since it involves the aforementioned side-conditions on unions.

#### **Lemma 7.23**

If $w_3\, {\mathrel {\bot }}\, w_1$ and $w_3\, {\mathrel {\bot }}\, w_2$ then:

$$\begin{aligned} w_3 \,{\mathrel {\cup }}\, w_1 = w_3 \,{\mathrel {\cup }}\, w_2 \quad \mathrm{implies}\quad w_1 = w_2. \end{aligned}$$

#### *Proof*

By induction on the derivations $w_3\, {\mathrel {\bot }}\, w_1$ and $w_3\, {\mathrel {\bot }}\, w_2$. We consider one case:Here, we have $\overline{w_3}\;{\mathbf {{b}}}_3 \,{\mathrel {\cup }}\, \overline{w_1}\;{\mathbf {{b}}}_1 = \overline{w_3}\;{\mathbf {{b}}}_3 \,{\mathrel {\cup }}\, {\mathbf {{b}}}_2$ by assumption, and therefore $\overline{w_1}\;{\mathbf {{b}}}_1 = {\mathbf {{b}}}_2$ by the cancellation law of a separation algebra. However, by assumption we also have $\lnot \mathsf{unmapped}\;(\overline{w_1}\;{\mathbf {{b}}}_1)$ and $\mathsf{unmapped}\;{\mathbf {{b}}}_2$, which contradicts $\overline{w_1}\;{\mathbf {{b}}}_1 = {\mathbf {{b}}}_2$.

#### **Definition 7.24**

The *separation algebra of memories* is defined as:
$P : \mathsf{mem}\rightarrow \mathsf{mem}\rightarrow \mathsf{index}\rightarrow \mathsf{Prop}$ and $f : \mathsf{mem}\rightarrow \mathsf{mem}\rightarrow \mathsf{index}\rightarrow \mathsf{option}\;\mathsf{mtree}$ are defined by case analysis on ${m_1}\,o$ and ${m_2}\,o$:The definitions of the omitted relations and operations are as expected.

The emptiness conditions ensure canonicity. Objects that solely consist of indeterminate bits with $\emptyset$ permission are meaningless and should not be kept at all. These conditions are needed for cancellativity.

#### **Fact 7.25**

If $$\Gamma ,\Delta \vdash m$$, then $$\mathsf{valid}\;m$$.

#### **Lemma 7.26**

If $$m_1\, {\mathrel {\bot }}\, m_2$$, then:

$$\begin{aligned} \Gamma ,\Delta \vdash m_1 \,{\mathrel {\cup }}\, m_2 \qquad \mathrm{iff}\qquad \Gamma ,\Delta \vdash m_1\hbox { and } \Gamma ,\Delta \vdash m_2. \end{aligned}$$

Notice that the memory typing environment $\Delta$ is not subdivided among $m_1$ and $m_2$. Consider the memory state corresponding to int x = 10, *p =&x:

Here, *w* is the memory tree that represents the integer value 10. The pointer on the right hand side is well-typed in the memory environment $\overline{\phantom {X}o_{\mathtt x} \mapsto w,\ o_{\mathtt p} \mapsto \bullet }$ of the whole memory, but not in $\overline{\phantom {X}o_{\mathtt p} \mapsto \bullet }$.

We prove some essential properties about the interaction between the separation algebra operations and the memory operations. These properties have been used in the soundness proof of the separation logic in the author’s PhD thesis [33].

#### **Lemma 7.27**

(Preservation of lookups) If $\Gamma ,\Delta \vdash m_1$ and , then:The relation  is part of a separation algebra, see Definition 5.1. We have  iff there is an $m_3$ with $m_1\, {\mathrel {\bot }}\, m_3$ and $m_2 = m_1 \,{\mathrel {\cup }}\, m_3$.

#### **Lemma 7.28**

(Preservation of disjointness) If $\Gamma ,\Delta \vdash m$ then:The relation  is defined in Definition 7.15. If , then each memory that is disjoint to *m* is also disjoint to $m'$.

As a corollary of the above lemma and Fact 7.16 we obtain that $m_1\, {\mathrel {\bot }}\, m_2$ implies disjointness of the memory operations:


#### **Lemma 7.29**

(Unions distribute) If $\Gamma ,\Delta \vdash m$ and $m_1\, {\mathrel {\bot }}\, m_2$ then:


Memory trees and memories can be generalized to contain elements of an arbitrary separation algebra as leaves instead of just permission annotated bits [32]. These generalized memories form a functor that lifts the separation algebra structure on the leaves to entire trees. We have taken this approach in the Coq development, but for brevity’s sake, we have refrained from doing so in this paper.

## Formalization in Coq

Real-world programming language have a large number of features that require large formal descriptions. As this paper has shown, the C programming language is not different in this regard. On top of that, the C semantics is very subtle due to an abundance of delicate corner cases. Designing a semantics for C and proving properties about such a semantics therefore inevitably requires computer support.

For these reasons, we have used Coq [15] to formalize all results in this paper. Although Coq does not guarantee the absence of mistakes in our definitions, it provides a rigorous set of checks on our definitions, for example by its type checking of definitions. On top of that, we have used Coq to prove all metatheoretical results stated in this paper. Last but not least, using Coq’s program extraction facility we have extracted an exploration tool to test our memory model on small example programs [33, 37]. Despite our choice to use Coq, we believe that nearly all parts of $\mathrm{CH}_2\mathrm{O}$ could be formalized in any proof assistant based on higher-order logic.

### Overloaded Typing Judgments

Type classes are used to overload notations for typing judgments (we have 25 different typing judgments). The class Valid is used for judgments without a type, such as $\vdash \Gamma$ and $\Gamma ,\Delta \vdash m$.
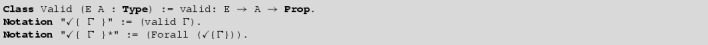


We use product types to represent judgments with multiple environments such as $\Gamma ,\Delta \vdash m$. The notation $\checkmark$ { $\Gamma$ } * is used to lift the judgment to lists. The class Typed is used for judgments such as $\Gamma ,\Delta \vdash v : \tau$ and .
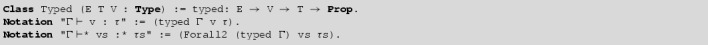


### Implementation-Defined Behavior

Type classes are used to parameterize the whole Coq development by implementation-defined parameters such as integer sizes. For example, Lemma 6.51 looks like:
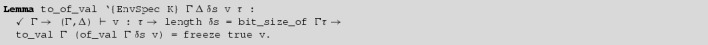


The parameter EnvSpec *K* is a type class describing an implementation environment with ranks *K* (Definition 4.12). Just as in this paper, the type *K* of integer ranks is a parameter of the inductive definition of types (see Definition 4.1) and is propagated through all syntax.
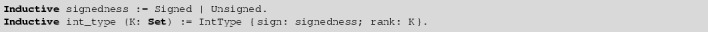


The definition of the type class EnvSpec is based on the approach of Spitters and van der Weegen [55]. We have a separate class Env for the operations that is an implicit parameter of the whole class and all lemmas.
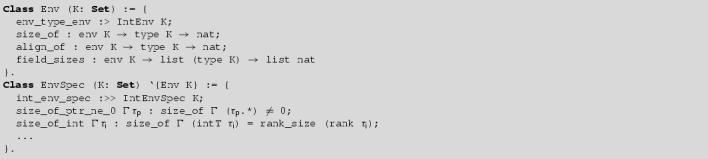


### Partial Functions

Although many operations in $\mathrm{CH}_2\mathrm{O}$ are partial, we have formalized many such operations as total functions that assign an appropriate default value. We followed the approach presented in Sect. 5.2 where operations are combined with a *validity predicate* that describes in which case they may be used. For example, part (2) of Lemma 7.29 is stated in the Coq development as follows:
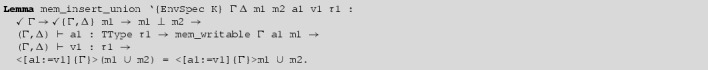


Here, m1 $\bot {}$ m2 is the side-condition of m1 $\cup {}$ m2, and mem_writable $\Gamma {}$ a1 m1 the side-condition of 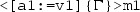. Alternatives approaches include using the option monad or dependent types, but our approach proved more convenient. In particular, since most validity predicates are given by an inductive definition, various proofs could be done by induction on the structure of the validity predicate. The cases one has to consider correspond exactly to the domain of the partial function.

Admissible side-conditions, such as in the above example 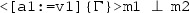 and 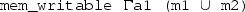, do not have to be stated explicitly and follow from the side-conditions that are already there. By avoiding the need to state admissible side-conditions, we avoid a blow-up in the number of side-conditions of many lemmas. We thus reduce the proof effort needed to use such a lemma.

### Automation

The proof style deployed in the $\mathrm{CH}_2\mathrm{O}$ development combines interactive proofs with automated proofs. In this section we describe some tactics and forms of proof automation deployed in the $\mathrm{CH}_2\mathrm{O}$ development.

*Small inversions* Coq’s inversion tactic has two serious shortcomings on inductively defined predicates with many constructors. It is rather slow and its way of controlling of names for variables and hypotheses is deficient. Hence, we often used the technique of small inversions by Monin and Shi [43] that improves on both shortcomings.

*Solving disjointness* We have used Coq’s setoid machinery [54] to enable rewriting using the relations  and  (Definition 7.15). Using this machinery, we have implemented a tactic that automatically solves entailments of the form:

$$\begin{aligned} H_0 : \bot \,{{x}}_0,\ \ldots ,\ H_n : \bot \,{{x}}_{n-1} \quad \vdash \quad \bot \,{{x}} \end{aligned}$$

where ${{x}}$ and ${{x}}_i$ (for $i < n$) are arbitrary Coq expressions built from $\emptyset$,  and $\bigcup$. This tactic works roughly as follows:

1. Simplify hypotheses using Theorem 7.18.
2. Solve side-conditions by simplification using Theorem 7.18 and a solver for list containment (implemented by reflection).
3. Repeat these steps until no further simplification is possible.
4. Finally, solve the goal by simplification using Theorem 7.18 and list containment.

This tactic is not implemented using reflection, but that is something we intend to do in future work to improve its performance.

*First-order logic* Many side-conditions we have encountered involve simple entailments of first-order logic such as distributing logical quantifiers combined with some propositional reasoning. Coq does not provide a solver for first-order logic apart from the firstorder tactic whose performance is already insufficient on small goals.

We have used Ltac to implemented an ad-hoc solver called naive_solver, which performs a simple breath-first search proof search. Although this tactic is inherently incomplete and suffers from some limitations, it turned out to be sufficient to solve many uninteresting side-conditions (without the need for classical axioms).

### Overview of the Coq Development

The Coq development of the memory model, which is entirely constructive and axiom free, consists of the following parts:

**Table:** 

Component	Sections	LOC
Support library (lists, finite sets, finite maps, *etc.*)	Section 2	12,524
Types and Integers	Section 4	1928
Permissions and separation algebras	Section 5	1811
Memory model	Section 6	8736
Refinements	Section 7.2	4046
Memory as separation algebra	Section 7.4	3844
Total		32,889

## Related Work

The idea of using a memory model based on trees instead of arrays of plain bits, and the idea of using pointers based on paths instead of offsets, has already been used for object oriented languages. It goes back at least to Rossie and Friedman [51], and has been used by Ramananandro et al. [48] for C++. Furthermore, many researchers have considered connections between unstructured and structured views of data in C [2, 14, 21, 56] in the context of program logics.

However, a memory model that combines an abstract tree based structure with low-level object representations in terms of bytes has not been explored before. In this section we will describe other formalizations of the C memory model.

*Norrish (1998)* Norrish has formalized a significant fragment of the C89 standard using the proof assistant HOL4 [44, 45]. He was the first to describe non-determinism and sequence points formally. Our treatment of these features has partly been based on his work. Norrish’s formalization of the C type system has some similarities with our type system: he has also omitted features that can be desugared and has proven type preservation.

Contrary to our work, Norrish has used an unstructured memory model based on sequences of bytes. Since he has considered the C89 standard in which effective types (and similar notions) were not introduced yet, his choice is appropriate. For C99 and beyond, a more detailed memory model like ours is needed, see also Sect. 3 and Defect Report #260 and #451 [26].

Another interesting difference is that Norrish represents abstract values (integers, pointers and structs) as sequences of bytes instead of mathematical values. Due to this, padding bytes retain their value while structs are copied. This is not faithful to the C99 standard and beyond.

*Leroy et al. (2006)* Leroy et al. have formalized a significant part of C using the Coq proof assistant [38, 39]. Their part of C, which is called CompCert C, covers most major features of C and can be compiled into assembly (PowerPC, ARM and x86) using a compiler written in Coq. Their compiler, called CompCert, has been proven correct with respect to the CompCert C and assembly semantics.

The goal of CompCert is essentially different from $\mathrm{CH}_2\mathrm{O}$’s. What can be proven with respect to the CompCert semantics does not have to hold for *any* C11 compiler, it just has to hold for the CompCert compiler. CompCert is therefore in its semantics allowed to restrict implementation defined behaviors to be very specific (for example, it uses 32-bit ints since it targets only 32-bit computing architectures) and allowed to give a defined semantics to various undefined behaviors (such as sequence point violations, violations of effective types, and certain uses of dangling pointers).

The CompCert memory model is used by all languages (from C until assembly) of the CompCert compiler [40, 41]. The CompCert memory is a finite partial function from object identifiers to objects. Each local, global and static variable, and invocation of malloc is associated with a unique object identifier of a separate object in memory. We have used the same approach in $\mathrm{CH}_2\mathrm{O}$, but there are some important differences. The paragraphs below discuss the relation of $\mathrm{CH}_2\mathrm{O}$ with the first and second version of the CompCert memory model.

*Leroy and Blazy (2008)* In the first version of the CompCert memory model [41], objects were represented as arrays of type-annotated fragments of base values. Examples of bytes are thus “the 2nd byte of the short 13” or “the 3rd byte of the pointer $(o,\,i)$”. Pointers were represented as pairs $(o,\,i)$ where *o* is an object identifier and *i* the byte offset into the object *o*.

Since bytes are annotated with types and could only be retrieved from memory using an expression of matching type, effective types on the level of base types are implicitly described. However, this does not match the C11 standard. For example, Leroy and Blazy do assign the return value 11 to the following program:
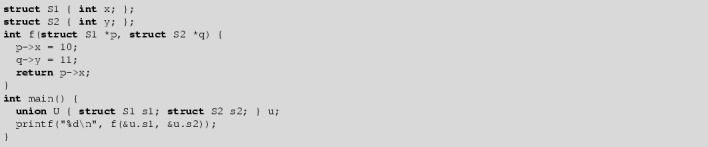


This code strongly resembles example [27, 6.5.2.3p9] from the C11 standard, which is stated to have undefined behavior.^6^ GCC and Clang optimize this code to print 10, which differs from the value assigned by Leroy and Blazy.

Apart from assigning too much defined behavior, Leroy and Blazy’s treatment of effective types also prohibits any form of “bit twiddling”.

Leroy and Blazy have introduced the notion of memory injections in [41]. This notion allows one to reason about memory transformations in an elegant way. Our notion of memory refinements (Sect. 7.2) generalize the approach of Leroy and Blazy to a tree based memory model.

*Leroy et al. (2012)* The second version of CompCert memory model [40] is entirely untyped and is extended with permissions. Symbolic bytes are only used for pointer values and indeterminate storage, whereas integer and floating point values are represented as numerical bytes (integers between 0 and $2^8-1$).

We have extended this approach by analogy to bit-representations, representing indeterminate storage and pointer values using symbolic bits, and integer values using concrete bits. This choice is detailed in Sect. 6.2.

As an extension of CompCert, Robert and Leroy have formally proven soundness of an alias analysis [50]. Their alias analysis is untyped and operates on the RTL intermediate language of CompCert.

Beringer et al. [7] have developed an extension of CompCert’s memory injections to reason about program transformations in the case of separate compilation. The issues of separate compilation are orthogonal to those that we consider.

*Appel et al. (2014)* The Verified Software Toolchain (VST) by Appel et al. provides a higher-order separation logic for Verifiable C, which is a variant of CompCert’s intermediate language Clight [3].

The VST is intended to be used together with the CompCert compiler. It gives very strong guarantees when done so. The soundness proof of the VST in conjunction with the correctness proof of the CompCert compiler ensure that the proven properties also hold for the generated assembly.

In case the verified program is compiled with a compiler different from CompCert, the trust in the program is still increased, but no full guarantees can be given. That is caused by the fact that CompCert’s intermediate language Clight uses a specific evaluation order and assigns defined behavior to many undefined behaviors of the C11 standard. For example, Clight assigns defined behavior to violations of effective types and sequence point violations. The VST inherits these defined behaviors from CompCert and allows one to use them in proofs.

Since the VST is linked to CompCert, it uses CompCert’s coarse permission system on the level of the operational semantics. Stewart and Appel [3, Chapter 42] have introduced a way to use a more fine grained permission system at the level of the separation logic without having to modify the Clight operational semantics. Their approach shows its merits when used for concurrency, in which case the memory model contains *ghost* data related to the conditions of locks [23, 24].

*Besson et al. (2014)* Besson et al. have proposed an extension of the CompCert memory model that assigns a defined semantics to operations that rely on the numerical values of uninitialized memory and pointers [8].

Objects in their memory model consist of lazily evaluated values described by symbolic expressions. These symbolic expressions are used to delay the evaluation of operations on uninitialized memory and pointer values. Only when a concrete value is needed (for example in case of the controlling expression of an if-then-else, for, or while statement), the symbolic expression is normalized. Consider:
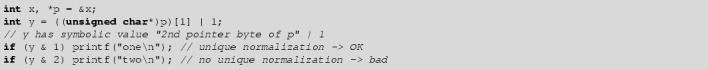


The value of 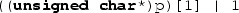 is not evaluated eagerly. Instead, the assignment to y stores a symbolic expression denoting this value. During the execution of the first if statement, the actual value of y & 1 is needed. In this case, 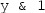 has the value 1 for any possible numerical value of ((unsigned char*)p)[1]. As a result, the string one is printed.

The semantics of Besson et al. is deterministic by definition. Normalization of symbolic expressions has defined behavior if and only if the expression can be normalized to a unique value under any choice of numeral values for pointer representations and uninitialized storage. In the second if statement this is not the case.

The approach of Besson et al. gives a semantics to some programming techniques that rely on the numerical representations of pointers and uninitialized memory. For example, it gives an appropriate semantics to pointer tagging in which unused bits of a pointer representation are used to store additional information.

However, as already observed by Kang et al. [28], Besson et al. do not give a semantics to many other useful cases. For example, printing the object representation of a struct, or computing the hash of a pointer value, is inherently non-deterministic. The approach of Besson et al. assigns undefined behavior to these use cases.

The goal of Besson et al. is inherently different from ours. Our goal is to describe the C11 standard faithfully whereas Besson et al. focus on *de facto* versions of C. They intentionally assign defined behavior to many constructs involving uninitialized memory that are clearly undefined according to the C11 standard, but that are nonetheless faithfully compiled by specific compilers.

*Ellison and Roşu (2012)* Ellison and Roşu [18, 19] have developed an executable semantics of the C11 standard using the $\mathbb {K}$-framework.^7^ Their semantics is very comprehensive and describes all features of a freestanding C implementation [27, 4p6] including some parts of the standard library. It furthermore has been thoroughly tested against test suites (such as the GCC torture test suite), and has been used as an oracle for compiler testing [49].

Ellison and Roşu support more C features than we do, but they do not have infrastructure for formal proofs, and thus have not established any metatheoretical properties about their semantics. Their semantics, despite being written in a formal framework, should more be seen as a debugger, a state space search tool, or possibly, as a model checker. It is unlikely to be of practical use in proof assistants because it is defined on top of a large C abstract syntax and uses a rather ad-hoc execution state that contains over 90 components.

Similar to our work, Ellison and Roşu’s goal is to *exactly* describe the C11 standard. However, for some programs their semantics is less precise than ours, which is mainly caused by their memory model, which is less principled than ours. Their memory model is based on CompCert’s: it is essentially a finite map of objects consisting of unstructured arrays of bytes.

*Hathhorn et al. (2015)* Hathhorn et al. [22] have extended the work of Ellison and Roşu to handle more underspecification of C11. Most importantly, the memory model has been extended and support for the type qualifiers const, restrict and volatile has been added.

Hathhorn et al. have extended the original memory model (which was based on CompCert’s) with decorations to handle effective types, restrictions on padding and the restrict qualifier. Effective types are modeled by a map that associates a type to each object. Their approach is less fine-grained than ours and is unable to account for active variants of unions. It thus does not assign undefined behavior to important violations of effective types and in turn does not allow compilers to perform optimizations based on type-based alias analysis. For example:
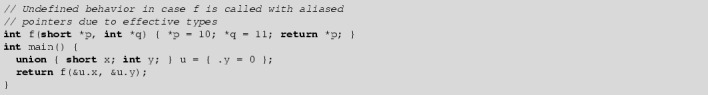


The above program has undefined behavior due to a violation of effective types. This is captured by our tree based memory model, but Hathhorn et al. require the program to return the value 11. When compiled with GCC or Clang with optimization level -O2, the compiled program returns the value 10.

Hathhorn et al. handle restrictions on padding bytes in the case of unions, but not in the case of structs. For example, the following program returns the value 1 according to their semantics, whereas it has unspecified behavior according to the C11 standard [27, 6.2.6.1p6] (see also Sect. 3.2):
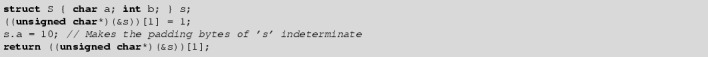


The restrictions on paddings bytes are implicit in our memory model based on structured trees, and thus handled correctly. The above examples provide evidence that a structured approach, especially combined with metatheoretical results, is more reliable than depending on ad-hoc decorations.

*Kang et al.* *(2015)* Kang et al. [28] have proposed a memory model that gives a semantics to pointer to integer casts. Their memory model uses a combination of numerical and symbolic representations of pointer values (whereas CompCert and $\mathrm{CH}_2\mathrm{O}$ always represent pointer values symbolically). Initially each pointer is represented symbolically, but whenever the numerical representation of a pointer is needed (due to a pointer to integer cast), it is non-deterministically *realized*.

The memory model of Kang et al. gives a semantics to pointer to integer casts while allowing common compiler optimizations that are invalid in a naive low-level memory model. They provide the following motivating example:
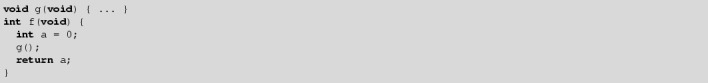


In a concrete memory model, there is the possibility that the function g is able to *guess* the numerical representation of &a, and thereby access or even modify a. This is undesirable, because it prevents the widely used optimization of constant propagation, which optimizes the variable a out.

In the CompCert and $\mathrm{CH}_2\mathrm{O}$ memory model, where pointers are represented symbolically, it is guaranteed that f has exclusive control over a. Since &a has not been leaked, g can impossibly access a. In the memory model of Kang et al. a pointer will only be given a numerical representation when it is cast to an integer. In the above code, no such casts appear, and g cannot access a.

The goal of Kang et al. is to give a unambiguous mathematical model for pointer to integer casts, but not necessarily to comply with C11 or existing compilers. Although we think that their model is a reasonable choice, it is unclear whether it is faithful to the C11 standard in the context of Defect Report #260 [26]. Consider:
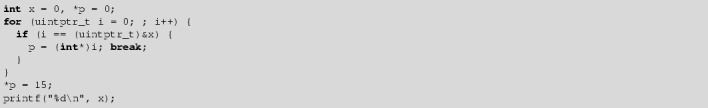


Here we loop through the range of integers of type uintptr_t until we have found the integer representation i of &x, which we then assign to the pointer p.

When compiled with gcc -O2 (version 4.9.2), the generated assembly no longer contains a loop, and the pointers p and &x are assumed not to alias. As a result, the program prints the old value of x, namely 0. In the memory model of Kang et al. the pointer obtained via the cast (int*)i is exactly the same as &x. In their model the program thus has defined behavior and is required to print 15.

We have reported this issue to the GCC bug tracker.^8^ However it unclear whether the GCC developers consider this a bug or not. Some developers seem to believe that this program has undefined behavior and that GCC’s optimizations are thus justified. Note that the cast (intptr_t)&x is already forbidden by the type system of $\mathrm{CH}_2\mathrm{O}$.

## Conclusion

In this paper we have given a formal description of a significant part of the non-concurrent C11 memory model. This formal description has been used in [33, 37] as part of an an operational, executable and axiomatic semantics of C. On top of this formal description, we have provided a comprehensive collection of metatheoretical results. All of these results have been formalized using the Coq proof assistant.

It would be interesting to investigate whether our memory model can be used to help the standard committee to improve future versions of the standard. For example, whether it could help to improve the standard’s prose description of effective types. As indicated on page 4 of Sect. 1, the standard’s description is not only ambiguous, but also does not cover its intent to enable type-based alias analysis. The description of our memory model is unambiguous and allows one to express intended consequences formally. We have formally proven soundness of an abstract version of type-based alias analysis with respect to our memory model (Theorem 7.2).

An obvious direction for future work is to extend the memory model with additional features. We give an overview of some features of C11 that are absent.

- *Floating point arithmetic* Representations of floating point numbers and the behaviors of floating point arithmetic are subject to a considerable amount of implementation defined behavior [27, 5.2.4.2.2].
  First of all, one could restrict to IEEE-754 floating point arithmetic, which has a clear specification [25] and a comprehensive formalization in Coq [10]. Boldo et al. have taken this approach in the context of CompCert [9] and we see no fundamental problems applying it to $\mathrm{CH}_2\mathrm{O}$ as well.
  Alternatively, one could consider formalizing all implementation-defined aspects of the description of floating arithmetic in the C11 standard.
- *Bitfields* Bitfields are fields of struct types that occupy individual bits [27, 6.7.2.1p9]. We do not foresee fundamental problems adding bitfields to $\mathrm{CH}_2\mathrm{O}$ as bits already constitute the smallest unit of storage in our memory model.
- *Untyped malloc* $\mathrm{CH}_2\mathrm{O}$ supports dynamic memory allocation via an operator $\mathsf{alloc}_{\tau }\;e$ close to C++’s new operator. The $\mathsf{alloc}_{\tau }\;e$ operator yields a $\tau {*}$ pointer to storage for a $\tau$-array of length *e*. This is different from C’s malloc function that yields a void* pointer to storage of unknown type [27, 7.22.3.4].
  Dynamic memory allocation via the untyped malloc function is closely related to unions and effective types. Only when dynamically allocated storage is actually used, it will receive an effective type. We expect one could treat malloced objects as unions that range over all possible types that fit.
- *Restrict qualifiers* The restrict qualifier can be applied to any pointer type to express that the pointers do not alias. Since the description in the C11 standard [27, 6.7.3.1] is ambiguous (most notably, it is unclear how it interacts with nested pointers and data types), formalization and metatheoretical proofs may provide prospects for clarification.
- *Volatile qualifiers* The volatile qualifier can be applied to any type to indicate that its value may be changed by an external process. It is meant to prevent compilers from optimizing away data accesses or reordering these [27, footnote 134]. Volatile accesses should thus be considered as a form of I/O.
- *Concurrency and atomics* Shared-memory concurrency and atomic operations are the main omission from the C11 standard in the $\mathrm{CH}_2\mathrm{O}$ semantics. Although shared-memory concurrency is a relatively new addition to the C and C++ standards, there is already a large body of ongoing work in this direction, see for example [4, 5, 52, 53, 57]. These works have led to improvements of the standard text.
  There are still important open problems in the area of concurrent memory models for already small sublanguages of C [4]. Current memory models for these sublanguages involve just features specific to threads and atomic operations whereas we have focused on structs, unions, effective types and indeterminate memory. We hope that both directions are largely orthogonal and will eventually merge into a fully fledged C11 memory model and semantics.

## Footnotes

1 The term *type-punning* merely appears in a footnote [27, footnote 95]. There is however the related *common initial sequence* rule [27, 6.5.2.3], for which the C11 standard uses the notion of *visible*. This notion is not clearly defined either.

2 See https://gcc.gnu.org/bugzilla/show_bug.cgi?id=61502.

3 The array size is ignored unless the static keyword is used. In case f would have the prototype , the pointer a should provide access to an array of at least 10 elements [27, 6.7.6.3]. The static keyword is not supported by $\mathrm{CH}_2\mathrm{O}$.

4 Contrary to traditional separation logic, we do not give local variables a special status of being *stack allocated*. We do so, because in C even local variables are allowed to have pointers to them.

5 Note that the C11 standard does not guarantee that the NULL pointer is represented as zeros, thus u.p is not necessarily NULL.

6 We have modified the example from the standard slightly in order to trigger optimizations by GCC and Clang.

7 This work has been superseded by Hathhorn et al. [22], which is described below.

8 See https://gcc.gnu.org/bugzilla/show_bug.cgi?id=65752.

## References

[1] Affeldt, R., Marti, N.: Towards formal verification of TLS network packet processing written in C. In: PLPV, pp. 35–46 (2013)

[2] Affeldt, R., Sakaguchi, K.: An intrinsic encoding of a subset of C and its application to TLS network packet processing. JFR. **7**(1), 63–104 (2014)

[3] Appel AW (2014). Program Logics for Certified Compilers.

[4] Batty, M., Memarian, K., Nienhuis, K. Pichon-Pharabod, J., Sewell, P.: The problem of programming language concurrency semantics. In: ESOP, volume 9032 of LNCS, pp. 283–307 (2015)

[5] Batty, M., Owens, S., Sarkar, S., Sewell, P., Weber, T.: Mathematizing C++ concurrency. In: POPL, pp. 55–66 (2011)

[6] Bengtson, J., Jensen, J.B., Sieczkowski, F., Birkedal, L.: Verifying Object-oriented programs with higher-order separation logic in Coq. In: ITP, volume 6898 of LNCS, pp. 22–38 (2011)

[7] Beringer, L., Stewart, G., Dockins, R., Appel, A.W.: Verified compilation for shared-memory C. In: ESOP, volume 8410 of LNCS, pp. 107–127 (2014)

[8] Besson, F., Blazy, S., Wilke, P.: A precise and abstract memory model for C using symbolic values. In: APLAS, volume 8858 of LNCS, pp. 449–468 (2014)

[9] Boldo, S., Jourdan, J.-H., Leroy, X., Melquiond, G.: A formally-verified C compiler supporting floating-point arithmetic. In: ARITH, pp. 107–115 (2013)

[10] Boldo, S., Melquiond, G.: Flocq: a unified library for proving floating-point algorithms in Coq. In: ARITH, pp. 243–252 (2011)

[11] Bornat, R., Calcagno, C., O’Hearn, P.W., Parkinson, M.J.: Permission accounting in separation logic. In: POPL, pp. 259–270 (2005)

[12] Boyland, J.: Checking interference with fractional permissions. In: SAS, volume 2694 of LNCS, pp. 55–72 (2003)

[13] Calcagno, C., O’Hearn, P.W., Yang, H.: Local action and abstract separation logic. In: LICS, pp. 366–378 (2007)

[14] Cohen E, Moskal M, Tobies S, Schulte W (2009). A precise yet efficient memory model for C. ENTCS.

[15] Coq Development Team: The Coq proof assistant reference manual. Available at https://coq.inria.fr/doc/ (2015)

[16] Dijkstra, E.W.: Cooperating sequential processes. In: Genuys, F. (ed.) Programming Languages: NATO Advanced Study Institute, pp. 43–112. Academic Press, Cambridge (1968)

[17] Dockins, R., Hobor, A., Appel, A.W.: A fresh look at separation algebras and share accounting. In: APLAS, volume 5904 of LNCS, pp. 161–177 (2009)

[18] Ellison, C.: A Formal Semantics of C with Applications. PhD thesis, University of Illinois (2012)

[19] Ellison, C., Roşu, G.: An executable formal semantics of C with applications. In: POPL, pp. 533–544 (2012)

[20] GCC: The GNU Compiler Collection. Website, available at http://gcc.gnu.org/

[21] Greenaway, D., Lim, J., Andronick, J., Klein, G.: Don’t sweat the small stuff: formal verification of C code without the pain. In: PLDI, pp. 429–439 (2014)

[22] Hathhorn, C., Ellison, C., Roşu, G.: Defining the undefinedness of C. In: PLDI, pp. 336–345 (2015)

[23] Hobor, A.: Oracle Semantics. PhD thesis, Princeton University, (2008)

[24] Hobor, A., Appel, A.W., Nardelli, F.Z.: Oracle semantics for concurrent separation logic. In: ESOP, volume 4960 of LNCS, pp. 353–367 (2008)

[25] IEEE Computer Society: 754-2008: IEEE Standard for Floating Point Arithmetic. IEEE (2008)

[26] ISO: WG14 Defect Report Summary. Website, available at http://www.open-std.org/jtc1/sc22/wg14/www/docs/

[27] ISO: ISO/IEC 9899-2011: Programming languages—C. ISO Working Group 14 (2012)

[28] Kang, J., Hur, C.-K., Mansky, W., Garbuzov, D., Zdancewic, S., Vafeiadis, V.: A formal c memory model supporting integer-pointer casts. In: PLDI, pp. 326–335 (2015)

[29] Klein, G., Kolanski, R., Boyton, A.: Mechanised separation algebra. In: ITP, volume 7406 of LNCS, pp. 332–337 (2012)

[30] Krebbers, R.: Aliasing restrictions of C11 formalized in Coq. In: CPP, volume 8307 of LNCS (2013)

[31] Krebbers, R.: An operational and axiomatic semantics for non-determinism and sequence points in C. In: POPL, pp. 101–112 (2014)

[32] Krebbers, R.: Separation algebras for C verification in Coq. In: VSTTE, volume 8471 of LNCS, pp. 150–166 (2014)

[33] Krebbers, R.: The C standard formalized in Coq. PhD thesis, Radboud University (2015)

[34] Krebbers, R., Leroy, X., Wiedijk, F.: Formal C semantics: CompCert and the C standard. In: ITP, volume 8558 of LNCS, pp. 543–548 (2014)

[35] Krebbers, R., Wiedijk, F.: A formalization of the C99 standard in HOL, Isabelle and Coq. In: CICM, volume 6824 of LNCS, pp. 297–299 (2011)

[36] Krebbers, R., Wiedijk, F.: Separation logic for non-local control flow and block scope variables. In: FoSSaCS, volume 7794 of LNCS, pp. 257–272 (2013)

[37] Krebbers, R., Wiedijk, F.: A typed C11 semantics for interactive theorem proving. In: CPP, pp. 15–27 (2015)

[38] Leroy, X.: Formal certification of a compiler back-end or: programming a compiler with a proof assistant. In: POPL, pp. 42–54 (2006)

[39] Leroy X (2009). Formal verification of a realistic compiler. CACM.

[40] Leroy, X., Appel, A.W., Blazy, S., Stewart, G.: The CompCert Memory Model, Version 2. Research report RR-7987, INRIA. Revised version available as Chapter 32 of [3] (2012)

[41] Leroy X, Blazy S (2008). Formal verification of a C-like memory model and its uses for verifying program transformations. JAR.

[42] Maclaren, N.: What is an Object in C Terms? Mailing list message. Available at http://www.open-std.org/jtc1/sc22/wg14/9350 (2001)

[43] Monin, J., Shi, X.: Handcrafted Inversions made operational on operational semantics. In: ITP, volume 7998 of LNCS, pp. 338–353 (2013)

[44] Norrish, M.: C formalised in HOL. PhD thesis, University of Cambridge (1998)

[45] Norrish, M.: Deterministic expressions in C. In: ESOP, volume 1576 of LNCS, pp. 147–161 (1999)

[46] O’Hearn, P.W.: Resources, concurrency and local reasoning. In: CONCUR, volume 3170 of LNCS, pp. 49–67 (2004)

[47] O’Hearn, P.W., Reynolds, J.C., Yang., H.: Local reasoning about programs that alter data structures. In: CSL, volume 2142 of LNCS, pp. 1–19 (2001)

[48] Ramananandro, T., Dos Reis, G., Leroy, X.: Formal verification of object layout for C++ multiple inheritance. In: POPL, pp. 67–80 (2011)

[49] Regehr, J., Chen, Y., Cuoq, P., Eide, E., Ellison, C., Yang, X.: Test-case reduction for C compiler bugs. In: PLDI, pp. 335–346 (2012)

[50] Robert, V., Leroy, X.: A formally-verified alias analysis. In: CPP, volume 7679 of LNCS, pp. 11–26 (2012)

[51] Rossie, J.G., Friedman, D.P.: An algebraic semantics of subobjects. In: OOPSLA, pp. 187–199 (1995)

[52] Sevcík J, Vafeiadis V, Nardelli FZ, Jagannathan S, Sewell P (2013). CompCertTSO: a verified compiler for relaxed-memory concurrency. JACM.

[53] Sewell P, Sarkar S, Owens S, Nardelli FZ, Myreen MO (2010). x86-TSO: a rigorous and usable programmer’s model for x86 multiprocessors. CACM.

[54] Sozeau, M.: A new look at generalized rewriting in type theory. JFR. **2**(1), 41–62 (2009)

[55] Spitters B, van der Weegen E (2011). Type classes for mathematics in type theory. Math. Struct. Comput. Sci..

[56] Tuch, H., Klein, G., Norrish, M.: Types, bytes, and separation logic. In: POPL, pp. 97–108 (2007)

[57] Vafeiadis, V., Balabonski, T., Chakraborty, S., Morisset, R., Nardelli, F.Z.: Common compiler optimisations are invalid in the C11 memory model and what we can do about it. In: POPL, pp. 209–220 (2015)

